# Perovskite nickelates as bio-electronic interfaces

**DOI:** 10.1038/s41467-019-09660-6

**Published:** 2019-04-10

**Authors:** Hai-Tian Zhang, Fan Zuo, Feiran Li, Henry Chan, Qiuyu Wu, Zhan Zhang, Badri Narayanan, Koushik Ramadoss, Indranil Chakraborty, Gobinda Saha, Ganesh Kamath, Kaushik Roy, Hua Zhou, Alexander A. Chubykin, Subramanian K. R. S. Sankaranarayanan, Jong Hyun Choi, Shriram Ramanathan

**Affiliations:** 1 School of Materials Engineering, Purdue University, West Lafayette, IN 47907 USA; 2 Lillian Gilbreth Fellowship Program, College of Engineering, Purdue University, West Lafayette, IN 47907 USA; 3 School of Mechanical Engineering, Purdue University, West Lafayette, IN 47907 USA; 4 Center for Nanoscale Materials, Argonne National Laboratory, Argonne, IL 60439 USA; 5 Department of Biological Sciences, Purdue Institute for Integrative Neuroscience, Purdue University, West Lafayette, IN 47907 USA; 6 X-ray Science Division, Advanced Photon Source, Argonne National Laboratory, Argonne, IL 60439 USA; 7 School of Electrical and Computer Engineering, Purdue University, West Lafayette, IN 47907 USA; 8 Present Address: Department of Chemistry and Physics, Indiana State University, Terre Haute, IN 47809 USA

## Abstract

Functional interfaces between electronics and biological matter are essential to diverse fields including health sciences and bio-engineering. Here, we report the discovery of spontaneous (no external energy input) hydrogen transfer from biological glucose reactions into SmNiO_3_, an archetypal perovskite quantum material. The enzymatic oxidation of glucose is monitored down to ~5 × 10^−16^ M concentration via hydrogen transfer to the nickelate lattice. The hydrogen atoms donate electrons to the Ni *d* orbital and induce electron localization through strong electron correlations. By enzyme specific modification, spontaneous transfer of hydrogen from the neurotransmitter dopamine can be monitored in physiological media. We then directly interface an acute mouse brain slice onto the nickelate devices and demonstrate measurement of neurotransmitter release upon electrical stimulation of the striatum region. These results open up avenues for use of emergent physics present in quantum materials in trace detection and conveyance of bio-matter, bio-chemical sciences, and brain-machine interfaces.

## Abstract (web summary)

Functional materials that act as bio-sensing media when interfaced with complex bio-matter are attractive for health sciences and bio-engineering. Here, the authors report room temperature enzyme-mediated spontaneous hydrogen transfer between a perovskite quantum material and glucose reactions.

## Introduction

Functional interfaces between biological and synthetic matter can greatly benefit from hydrogen transfer, which is of broad relevance to bio-sensing and bio-chemical sciences. Sensing media that responds to low concentrations of bio-markers therefore can be relevant in this context, however, must be functional near room (or body) temperature while constantly exposed to complex biological media. As a promising candidate, the perovskite nickelate SmNiO_3_ (SNO, space group Pbnm)^1^, is water-stable, and belongs to a class of strongly correlated quantum materials, whose properties are highly sensitive to the occupancy of electrons in their partially filled orbitals^2–4^. When doped with charge carriers, SNO shows massive electronic structure changes: For one electron/unit cell doping from hydrogen, the electrical resistance changes by ~10 orders of magnitude^5^. In previous work, perovskite nickelates have shown potential for electric field detection in salt water media^6^. Glucose is a sugar that is essential for energy production in organisms and widely serves as a model system for bio-chemical studies. In nature, glucose can be oxidized into gluconolactone by losing hydrogen in the presence of glucose oxidase (GOx) enzyme^7^, and this reaction is seen across various organisms^8,9^. Utilizing an external electric field, perovskite oxide nano-particles have been used for glucose detection^10,11^. An important strategy to understand such biological and bio-chemical reactions involves measurement of the hydrogen transfer processes. Here, we present enzyme-mediated spontaneous hydrogen transfer between glucose reaction and SNO devices, as well as interfacing perovskite devices with acute mouse brain slices.

## Results

### Reaction mechanism

**Fig. 1:**
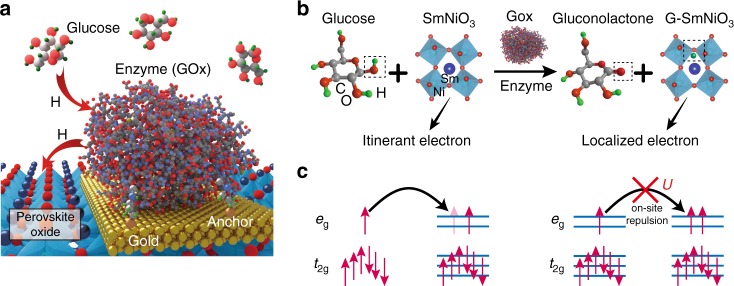
Spontaneous hydrogen transfer between perovskite and glucose–enzyme reaction. **a** Schematic figure of the atomic hydrogen transfer from the glucose to perovskite. The glucose oxidase (GOx) enzymes are anchored on the gold electrode via cystamine bonding (details are described in Supplementary Fig. 1). Figure not drawn to scale for clarity. **b** Reaction mechanism of glucose+SmNiO_3_ transformation to gluconolactone+G-SmNiO_3_. The GOx enzyme serves as a catalyst and transfers hydrogen from glucose to SmNiO_3_, referred to as G-SmNiO_3_. The hydrogens bonded with carbons are omitted for figure clarity. **c** The electron filling configuration of the Ni 3*d* orbitals in SmNiO_3_ and G-SmNiO_3_. For the pristine SmNiO_3_, the *e*_*g*_ orbitals are singly occupied. In the case of G-SmNiO_3_, the donors doped from the hydrogen occupy an *e*_*g*_ orbital, resulting in large on-site columbic repulsion energy *U*, and localizing the charge carriers resulting in reduction of electronic conductivity

Figure 1a shows the schematic pathway for spontaneous atomic hydrogen transfer between glucose–GOx reaction and a perovskite, where the nickelate participates in the reaction by accepting the hydrogen in the glucose–enzyme–oxide transfer chain. The reaction mechanism is described in Fig. 1b. During the glucose–enzyme–SNO reaction, the hydrogen atoms from the glucose are first transferred to the GOx enzyme as it occurs in nature, and then into the SNO lattice. This process occurs spontaneously without the need for any external energy input. The hydrogen then bonds with oxygen anions and occupies interstitial sites among the oxygen octahedra in SmNiO_3_, contributing an electron to the *d* orbitals of nickel^5^. The hydrogen acts as a donor dopant in the lattice. As a result, the singly occupied Ni *e*_*g*_ orbitals in glucose-reacted SNO (GSNO) become doubly occupied and the additional electron in the *e*_*g*_ orbital imposes large on-site Mott–Hubbard electron–electron repulsion, leading to localization of the charge carriers and resistivity increase^1,12^, as shown in Fig. 1c. Such a hydrogen-induced conduction suppression serves as a sensitive platform for chemical transduction at the interface between the nickelate films and biological glucose reaction.

### Electrical characterization

**Fig. 2:**
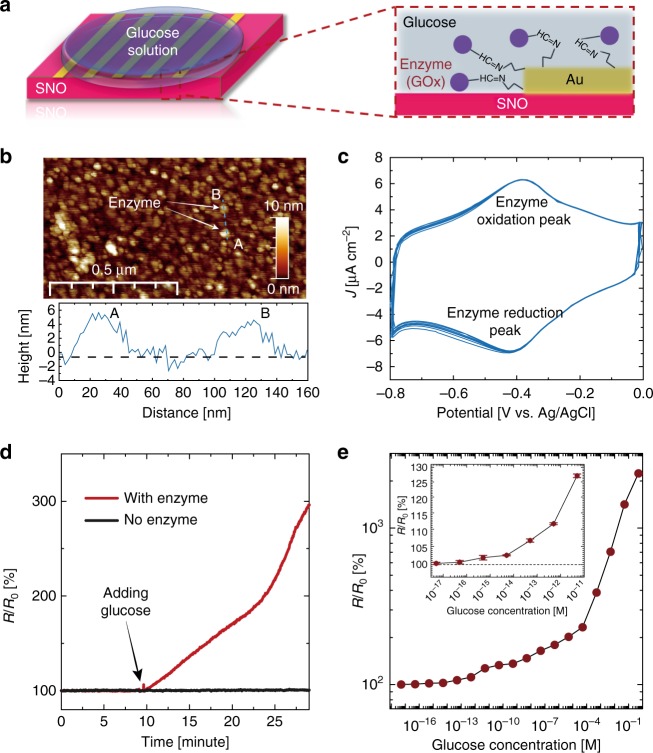
Electrical response of nickelate devices interfaced with glucose without external energy. **a** Schematic figure of the enzyme-SmNiO_3_ (SNO) device, with glucose oxidase (GOx) decorated Au electrodes. Before the reaction, glucose solution was added on top of the device surface, as shown in the zoomed in figure on the right. **b** The surface morphology of GOx-modified Au surface measured by atomic force microscopy (AFM). The GOx molecules are the bright dots on the surface and a line scan along AB shows the height of the GOx is around 4–5 nm. **c** Cyclic voltammetry (CV) measurements with the GOx-modified Au surface as a working electrode. Electrochemical reduction and oxidation peaks of GOx were observed as expected^14^. **d** Temporal resistance of the enzyme–SNO device after 0.5 M glucose solution is applied as shown in (**a**). A clear increase in resistance is observed after the glucose solution is applied (red curve). No change in resistance was observed for the control SNO sample without any GOx modification (black curve in the inset). *R*_0_ is the resistance of the pristine enzyme–SNO device. **e** Resistance increase of the enzyme–SNO device after the device is soaked in glucose solution for 1 h with different concentration. A monotonic increase of *R*/*R*_0_ is observed with increasing glucose concentration. The enzyme–SNO device is responsive to glucose concentration down to 5 × 10^−16^ M (signal to noise ratio >3). The error bar shown in inset plot was determined from the standard deviation of 10 measurements

To demonstrate the hydrogen transfer from the glucose–GOx reaction to SNO, SNO devices with GOx-modified Au electrodes were first fabricated, as schematically shown in Fig. 2a (for details, see Supplementary Methods and Supplementary Fig. 1). Next, atomic force microscopy (AFM) and cyclic voltammetry (CV) measurements were performed to verify the successful decoration of GOx on Au surface. As shown in Fig. 2b, bright GOx dots were observed on the Au surface. A line scan along AB indicates the height of GOx is around 5 nm, which is consistent with the actual size of GOx^13^. The pristine Au surface is smooth with a roughness of ~0.7 nm (Supplementary Fig. 2). In the CV scan, a pair of reversible electron transfer peaks were observed at the position characteristic of the GOx enzyme (Fig. 2c)^14^. No CV peak was found at this voltage region when the measurement is performed on a bare Au electrode surface (Supplementary Fig. 3). With these measurements, we can confirm the existence of GOx on the Au surface. The reaction between the enzyme–SNO device and glucose solution was initiated by applying a droplet (20 μL) of 0.5 M glucose solution (in deionized water (DI) water) on top of the device, as schematically shown in Fig. 2a. After the glucose droplet was applied, a sharp increase of resistance of the enzyme–SNO device was observed, see the red curve in Fig. 2d. However, if there is no GOx decoration on the SNO device, no reaction occurs between the glucose solution and the nickelate device, as shown in the black curve of Fig. 2d. The reacted solution was subsequently characterized by Fourier-transform infrared (FTIR) spectroscopy measurement, and the formation of gluconolactone was observed (Supplementary Fig. 4), which is consistent with the reaction mechanism described in Fig. 1b. The reaction occurs spontaneously without any external electric fields (Supplementary Fig. 5). The resistance of the device can be reversed back to original state by annealing, due to the room temperature metastable trapping of the hydrogen in the perovskite lattice^5^. After the recovery, new GOx enzyme can be decorated to the same device and the entire process can be reproduced (Supplementary Fig. 5).

To demonstrate the crucial role of SNO in this reaction, GOx were used to modify Au electrodes on control groups, including transparent oxide conductors such as indium-doped SnO_2_ (ITO), fluorine-doped SnO_2_ (FTO), and Pd, an elemental metal. No change in electrical behavior was observed (Supplementary Fig. 6). SrTiO_3_ and Nb-doped SrTiO_3_ with empty and partially filled *d* orbitals were also decorated with GOx and no spontaneous hydrogen transfer was seen (Supplementary Fig. 7). The SNO devices were stable in water and the doping from glucose was non-volatile at room temperature (Supplementary Figs. 8 and 9). The enzyme–SNO devices were highly responsive to dilute glucose concentrations and showed good selectivity. For the responsivity test, the enzyme–SNO devices were soaked in glucose solution with different concentrations for one hour and then the resistance ratio (*R*/*R*_0_) was plotted in Fig. 2e. In all the cases, the device resistance increased after the reaction, and *R*/*R*_0_ becomes larger with increasing glucose concentration. The *R*/*R*_0_ at the dilute limit of the glucose concentration is shown in the inset of Fig. 2e and the detection limit is determined as 5 × 10^−16^ M (signal to noise ratio >3) (for comparison with glucose sensing literature, see Supplementary Fig. 10). The high detection limit in our enzyme–SNO devices is a unique attribute of strong electron correlations, a quantum mechanical effect wherein miniscule perturbation to the electron occupancy of orbitals can result in giant modulation of the transport gap^1^. The detection of glucose is reproducible as shown in Supplementary Fig. 11. The GOx–SNO devices also function at body temperature (37 °C), see Supplementary Fig. 12. To test the selectivity of the enzyme–SNO device, 20 μL of 0.5 M mannose, galactose and glucose solutions were separately applied to the enzyme–SNO device, no reaction was observed for the mannose and galactose solution as seen from electrical characterization (Supplementary Fig. 13).

### Synchrotron X-ray-based characterization

**Fig. 3:**
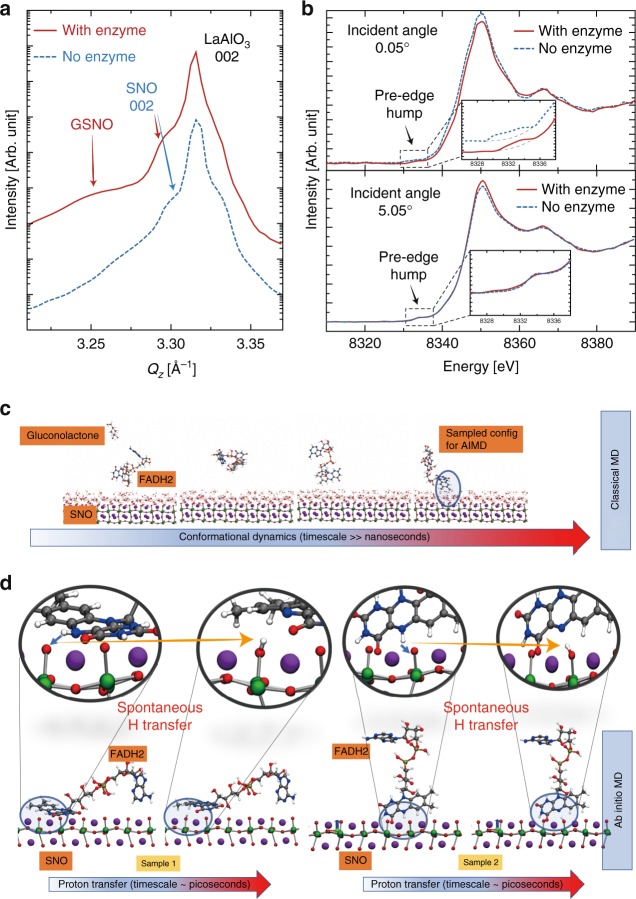
Mechanism of the spontaneous reaction between the enzyme–SNO device and glucose. **a** Synchrotron X-ray diffraction scans of glucose-reacted SmNiO_3_ (SNO) devices with and without glucose oxidase (GOx) enzyme modification. The scans are along *Q*_*z*_ direction around the 002 peak of LaAlO_3_ substrate (pseudocubic notation). **b** Angle-dependent X-ray absorption near edge spectroscopy (XANES) spectra on glucose-reacted SNO devices with and without GOx enzyme modification (Ni K-edge). At a surface sensitive incident angle of 0.05^o^, XANES spectra acquired on the GOx-modified electrode on GSNO show pronounced reduction in the white line peak amplitude and the effective pre-edge humps, as compared to the electrode without any GOx, suggesting orbital filling at the SNO surface, due to the hydrogen transfer. The blue dashed curve in the figure inset is shifted upward for clarity of data presentation. At incidence angle of 5.05^o^, XANES spectra acquired on GOx-modified device shows negligible difference with respect to that without enzyme modification, which indicates the majority of the film is still pristine SNO. The insets show zoomed-in pre-edge feature in XANES spectra. **c** Classical MD trajectory of a representative FADH2 molecule. Snapshots show the conformational changes that the FADH2 molecule undergoes over timescales of ~10 ns before approaching the SNO (001) surface (pseudocubic notation). **d** Several tens of FADH2 near-surface conformations from ~500 ns of classical MD trajectories are sampled and used as starting configurations for AIMD simulations. Two representative samples are illustrated to demonstrate the spontaneous hydrogen transfer from an H site in FADH2 to surface oxygen of SNO (001). In both the depicted cases, one of the hydrogens from FADH2 gets extracted and gets adsorbed into the SNO (001) (zoomed-in view); the extraction process is spontaneous, with an energetic gain as large as 1.8 eV. Classical MD simulations suggest that the steric effects are important and can hinder the hydrogen transfer from FADH2 to SNO (001) as shown by detailed first principles calculations of representative trajectories (see Supplementary Fig. 21)

X-ray diffraction measurements were performed to study the structural evolution in glucose reacted nickelates (GSNO) with scans around LaAlO_3_ substrate 002 peak (pseudocubic notation). The sample abbreviation and treatment conditions are summarized in Supplementary Table 1. The pristine SNO 002 peak was observed with a lower *Q*_*z*_ value compared to the LaAlO_3_ substrate due to its larger out of plane lattice parameter, see Supplementary Fig. 14. Figure 3a shows position-specific x-ray diffraction data of the reacted GSNO sample with patterned electrodes. Red solid curve is on top of a GOx-modified Au electrode, while blue dashed curve is on a Pd electrode without any GOx modification. After the reaction, an extra peak with smaller *Q*_*z*_ (arising from the hydrogen doping-induced lattice expansion^6^) was found in the red curve besides the original SNO 002 peak, while no extra peak was seen for the sample without enzyme modification. The observation of both pristine and hydrogen-doped SNO peaks in the red curve suggests a two-layer structure, where the hydrogen-doped SNO layer is restricted to a thin near-surface layer on top of the pristine SNO due to the self-limiting kinetics at room temperature and the fact there is no external energy supplied.

A combination of angle-dependent X-ray absorption near edge spectroscopy (XANES) measurements and electron transport modeling was performed to investigate the depth profile of hydrogen in the GSNO. Figure 3b show angle-dependent XANES spectra (Ni K-edge), presenting a significant contrast between the surface of GSNO and deeper layers in the film. At the incident angle (0.05°) below the total reflection critical angle that entails surface sensitive measurements, the XANES spectra on respective Au (GOx modified) and Pd (no GOx) electrodes show pronounced differences. Firstly, the white line peak amplitude explicitly gets weaker on GOx-modified Au electrode as compared to that on Pd electrode. Secondly, the effective integrated area underneath the XANES pre-edge hump exhibits significant reduction on GOx-modified Au electrode. Both reductions indicate *d*-orbital filling due to the electron doping at the near-surface region. In large contrast, the XANES spectra overlap in all aspects between the electrodes with and without enzyme at large angle of incidence (5.05°). The reduction in the pre-edge hump area at different incidence angles is quantified by the area ratio between pristine SNO (no enzyme) and GSNO (with enzyme). For the 0.05° incidence angle, the area ratio is 2.60, suggesting hydrogen doping at the surface. Almost no reduction was found for 5.05° incidence angle, with a ratio of 1.04. The shallow X-ray probing depth at an incidence angle of 0.05° sets the maximum (upper bound) doping layer thickness to ~10 nm. Tunneling transport modeling of the glucose-treated SNO devices in fact indicates the doped layer to be of the order of 1 nm thickness (Supplementary Figs. 15 and 16). While the self-limiting kinetics at near room temperature eventually leads to a thin fully doped surface layer, the GOx–SNO devices can be used numerous times before the resistance saturation is reached, as shown in Supplementary Fig. 17.

### Classical and quantum mechanical simulations

We use a combination of classical molecular dynamics (MD) and quantum chemical simulations to understand the thermodynamics and kinetics of the spontaneous hydrogen transfer mechanism. There are two key steps involved: the first reductive half-reaction of β-d-glucose to gluconolactone happens spontaneously in presence of GOx enzyme, where 1,3 hydroxyl groups of glucose donate hydrogen to the redox cofactor flavin adenine dinucleotide (FAD) of GOx, forming FADH2. This process has been studied in quantum chemical and docking simulations, with a heat of formation ~−600 kcal/mol^15,16^. The second step involves hydrogen transfer from FADH2 to the strongly correlated oxide SNO. We evaluate the energetics of dehydrogenation of FADH2 using quantum chemical simulations. While the energetic cost to dehydrogenate FADH2 is high (~2.2–3.2 eV/hydrogen), the presence of SNO allows for spontaneous hydrogen transfer from FADH2 to SNO (see Supplementary Fig. 18 and Supplementary Methods for details).

To simulate the dynamics of FADH2 interaction with SNO, we perform classical MD simulations to adequately sample sterically acceptable configurations of FADH2 at the active SNO sites, i.e. surface O (see the simulation box in Supplementary Fig. 19). The classical MD simulations suggest that the conformational dynamics of FADH2 is a slow process and the diffusion of FADH2 molecules to the SNO (001) (pseudocubic notation) surface occurs at timescales of tens of nanosecond (see Fig. 3c for snapshots from a representative trajectory and Supplementary Movie 1). The FADH2 molecules undergo a series of conformational changes before adsorbing onto the SNO (001) surface. We sample several such energetically favorable near surface configurations of FADH2 from the classical MD and use it as starting configurations for smaller ab initio MD (AIMD) models to study the effects of strong correlation and its role in FADH2 dehydrogenation (see Supplementary Fig. 20). Figure 3d shows snapshots from two representative AIMD trajectories that depict the temporal evolution of the FADH2 molecules near the SNO surface. The magnified images track the FADH2 and the NiO_6_ octahedra near the SNO surface (top panel of Fig. 3d). For both the cases shown, we observe spontaneous hydrogen transfer to surface oxygen of SNO within 2 ps of simulation, also see Supplementary Movie 2. This picture is consistent with the enzyme-assisted hydrogen transfer mechanism depicted schematically in Fig. 1. We find that the conformations of the FADH2 play a key role in dictating the hydrogen transfer: If the FADH2 conformations are sterically favorable, the process is spontaneous (see Supplementary Fig. 21 and Supplementary Movie 3).

### Interfacing with mouse brain slice

**Fig. 4:**
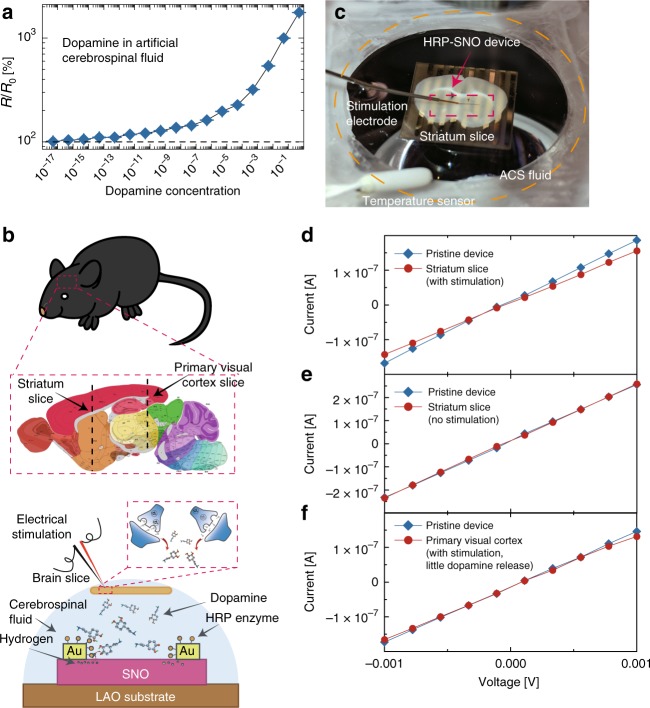
Direct interfacing of HRP–SNO device with acute mouse brain slice. **a** Electrical response of the horseradish peroxidase–SmNiO_3_ (HRP–SNO) devices to varying dopamine concentration in artificial cerebrospinal fluid. The device resistance change is presented as ratio before and after the reaction (*R*/*R*_0_). The error bar was determined from the standard deviation of 10 measurements in each case. **b** A schematic (drawing not to scale for clarity) showing the process of interfacing acute mouse brain slice with the HRP–SNO device. The black dash lines in the brain anatomy map show where the striatum slice and primary visual cortex slice were cut. Under electrical stimulation, dopamine molecules are released from the striatum slice and dope the SNO device through the hydrogen transfer assisted by the HRP enzyme. The brain anatomy image is adapted with permission from an open data resource © 2015 Allen Institute for Brain Science. Allen Brain Atlas API^26^. Available from: http://mouse.brain-map.org/. **c** A photo of the experimental set up during the interfacing between striatum slice and HRP–SNO device. The experiment was performed in an aqueous artificial cerebrospinal (ACS) fluid environment and the stimulation electrode was used to trigger dopamine release from the striatum slice. The striatal brain slice is ~10 × 5 mm and the HRP–SNO device region (red rectangle) is fully covered under the slice. **d** *I*–*V* characteristics of the HRP–SNO device interfaced with striatal brain slice. When stimulated, the striatal brain slice releases dopamine which can be monitored by the HRP–SNO devices as seen from change in channel resistance. **e** The HRP–SNO device was interfaced with striatum slice in the same way as described in Fig. 4c, but with no electrical stimulation (and thus no dopamine release). No resistance change was seen, and the device was stable in the spinal fluid environment. **f** The primary visual cortex part of the mouse brain which releases little or no dopamine under electrical stimulation[24] was interfaced with the HRP–SNO device, After the electrical stimulation, much smaller response (only ~2% change in resistance) was observed compared to that of striatum slice stimulation

We further extended the experimental studies to another important bio-marker dopamine (DA), which is a neurotransmitter that plays a significant role in motivation and learning^17^. Low levels of DA are causal to the progression of Parkinson’s disease (PD), and are hypothesized to be implicated in schizophrenia and attention deficit hyperactivity disorder (ADHD)^18–20^. Consequently, detection of low concentrations of DA is required for future studies of these diseases and for the development of pharmacological therapies^21^. DA can be monitored by our nickelate devices using the horseradish peroxidase (HRP) enzyme, as schematically shown in Supplementary Fig. 22a. The HRP–SNO device is responsive to DA both in DI water down to 5 × 10^−17^ M (Supplementary Fig. 22b and Supplementary Fig. 23 for comparison with literature). The HRP–SNO devices were also functional in biological media and responded to DA in artificial cerebrospinal fluid (ACSF) (see Fig. 4a). As control experiments, the HRP–SNO device was found to be stable in both pure ACSF and DI water, and the HRP enzyme is essential for the hydrogen transfer process to the nickelate lattice (Supplementary Fig. 24). Enzymatic selectivity coupled with the spontaneous ion–electron transfer therefore ensures robustness of the nickelate quantum material in various biological and brain environments.

We then directly interfaced an acute mouse brain slice onto the nickelate devices to monitor DA release triggered by electrical stimulation of the striatum, the brain area enriched with dopaminergic projections, as schematically shown in Fig. 4b and c. In this experiment, an acute mouse striatal slice was placed on a HRP–SNO device in a chamber continuously perfused with oxygenated ACSF solution (see the Supplementary Methods section for complete details), and electrical stimulation was applied to trigger the release of DA from the striatum^22^. Figure 4d shows the corresponding response of the HRP–SNO device to DA released from stimulated striatal slice. The resistance increase of the HRP–SNO device (~23%) approximately corresponds to DA concentration of 10^−10^–10^−9^ M, based on the DA-concentration-dependent experiments shown in Fig. 4a. Such an estimation is consistent with stimulation experiments under similar conditions^23^, considering that only a small fraction of the DA molecules effuse out from the brain synapses and reach the HRP–SNO device surface. As a control experiment, the HRP–SNO device was interfaced with a striatal slice without electrical stimulation, and negligible response was observed (Fig. 4e). In another control experiment, identical electrical stimulation was applied to a primary visual cortex (V1) slice where there is expected to be little or no DA innervation, and therefore minimal or no DA was expected to be released^24^. A much smaller response (only ~2% resistance change) was found from the HRP–SNO device interfaced with stimulated V1 slice compared to the case of stimulated striatal slice, suggesting the large response observed with striatum slice stimulation is from DA release (see Fig. 4f). The much smaller response observed with V1 stimulation is likely from small amounts of DA-like species such as serotonin^25^. Also, the HRP enzyme was found to be critical in transferring the hydrogen from DA to SNO. No change in resistance was found when the SNO device with only gold electrodes (without HRP enzyme) was interfaced with the striatal slices while the same electrical stimulation was applied (see Supplementary Fig. 25).

## Discussion

We have presented the discovery of room temperature enzyme-mediated spontaneous hydrogen transfer from model biological reactions and brain matter into a perovskite quantum material. The hydrogen transfer from biological reactions at the nickelate interface trigger a unique response: strong Coulomb repulsion that localizes charge carriers and suppresses electrical conduction. Coupled with the ability to function at body temperature in brain and biological environments enables response to ultra-low concentrations of bio-markers. The results open up directions for exploring correlated quantum systems in health sciences, brain interfaces and biological routes to dope emerging semiconductors.

### Reporting summary

Further information on experimental design is available in the Nature Research Reporting Summary linked to this article.

## Supplementary information

Supplementary Information

Description of Additional Supplementary Files

Supplementary Movie 1

Supplementary Movie 2

Supplementary Movie 3

Reporting Summary

## Data Availability

All data presented in the main text and the supplementary information are available.
