# Supplementary material for: Perovskite nickelates as bio-electronic interfaces
Source: Nat Commun. 2019 Apr 10;10:1651. doi: 10.1038/s41467-019-09660-6 (PMC6458181; doi:10.1038/s41467-019-09660-6)
Supplement: Supplementary file 1 — Supplementary Information [file 41467_2019_9660_MOESM1_ESM.pdf]

Supplementary information

Perovskite nickelates as bio-electronic interfaces

Zhang *et al*

This file includes:

- Supplementary Figures
- Supplementary Tables
- Supplementary Methods
- Supplementary References

Supplementary Figures

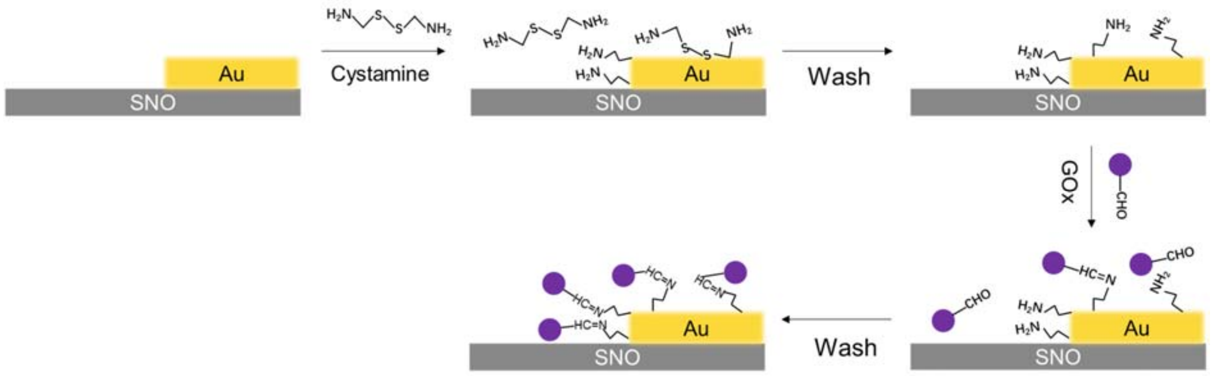

Supplementary Figure 1. Process flow for decorating GOx on the Au surface. As detailed in the experimental methods section, cystamine was first used to modify the Au surface by forming S-Au bonds. Then the NH_2 groups on the Au surface react with the CHO groups in oxidized GOx, after which the GOx molecules were chemically attached on the Au surface.

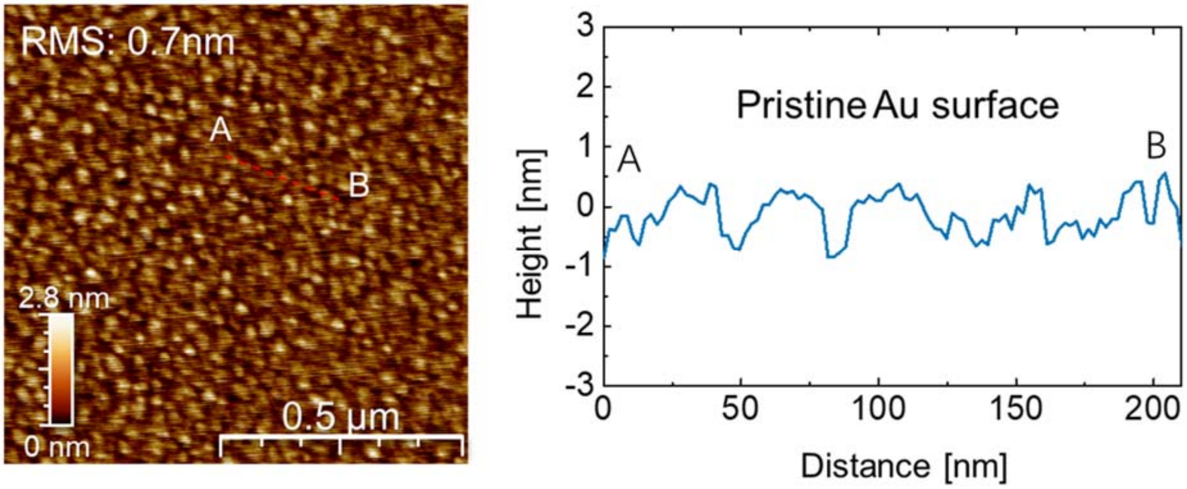

Supplementary Figure 2. Morphology of the pristine Au surface before GOx modification. Before GOx modification, the AFM scan shows a smooth Au surface with a roughness of ~0.7 nm. A line scan along AB shows a smooth topography.

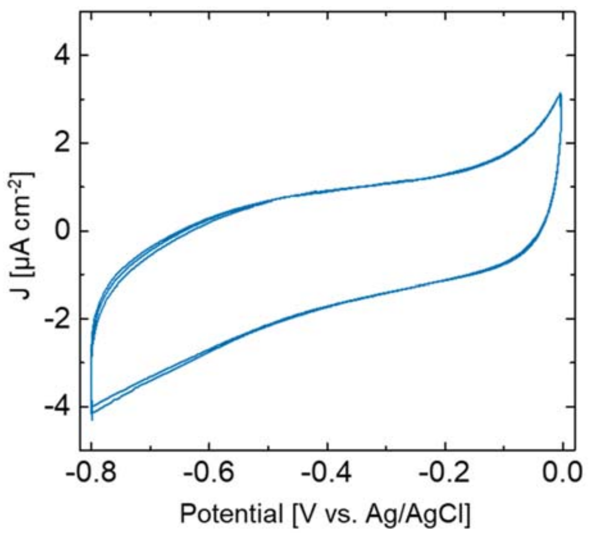

Supplementary Figure 3. CV measurement of the pristine Au surface *before* GOx decoration. CV measurements were performed on a control sample of Au without any GOx modification. No GOx electrochemical reaction peak is present as to be expected.

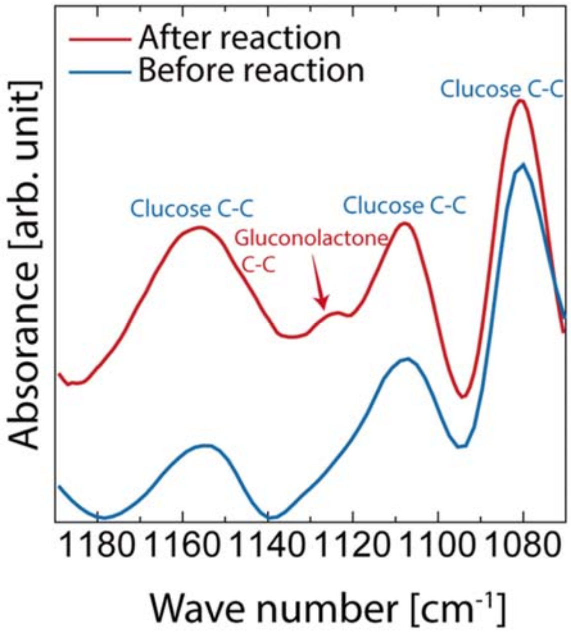

Supplementary Figure 4. FTIR measurement of the glucose solution reacted with the GOx-SNO device. Before the reaction (blue curve), Fourier-transform infrared (FTIR) spectroscopy measurement results show only the glucose vibration peaks. After the reaction (red curve), an extra peak was noticed near wavenumber 1120 cm^{-1}, whose location is consistent with the characteristic peak of the gluconolactone.[1,2] The FTIR experiments confirm the formation of gluconolactone after the reaction between the GOx-SNO device and glucose solution, which agrees with the proposed reaction mechanism.

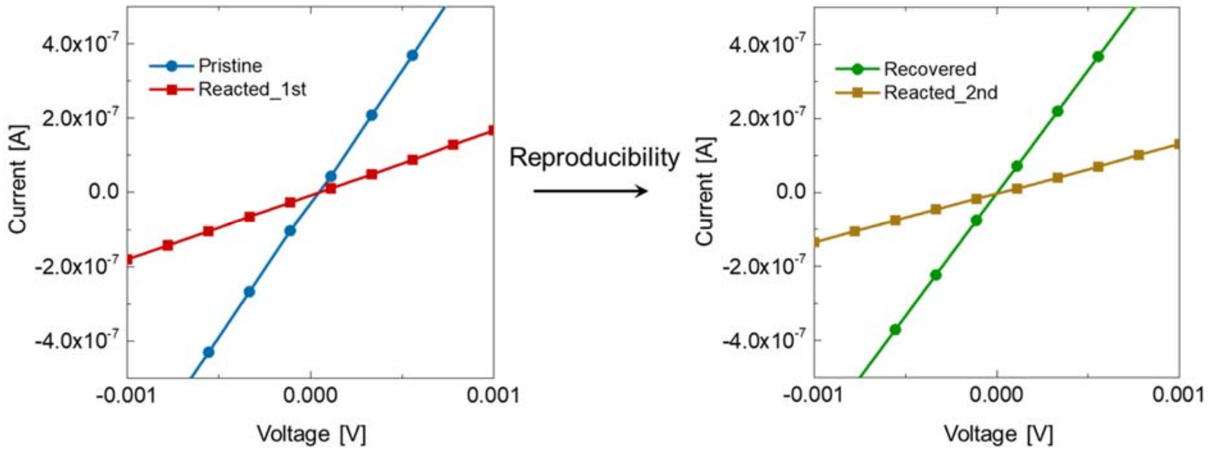

Supplementary Figure 5. Spontaneous reaction and re-use of the tested device. To demonstrate that the hydrogen transfer reaction occurs without any external electric field, the GOx-SNO device was soaked in 0.5 M glucose solution for 20 minutes. Then the resistance of the device was measured and corresponding increase of the SNO resistance was observed (red curve). The device was then annealed in air at 200 °C for 5 minutes to recover to near the pristine state (green curve). This recovery behavior is due to the metastable anchoring of the dopant in the lattice.[3,4] By re-decorating the device with new GOx, the reaction between SNO-GOx device and glucose can be reproduced (brown curve).

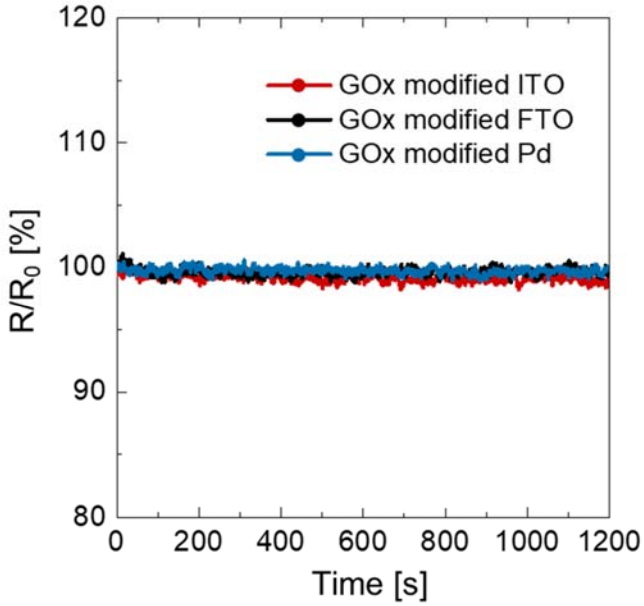

Supplementary Figure 6. Absence of reaction between glucose and GOx modified ITO, FTO and Pd. To demonstrate the necessity of SNO in the observed reaction, Au electrodes on conducting oxides, e.g. indium-doped SnO_2 (ITO), fluorine-doped SnO_2 (FTO), as well as elemental Pd were modified with GOx. After glucose solution application, the resistance of these devices did not change.

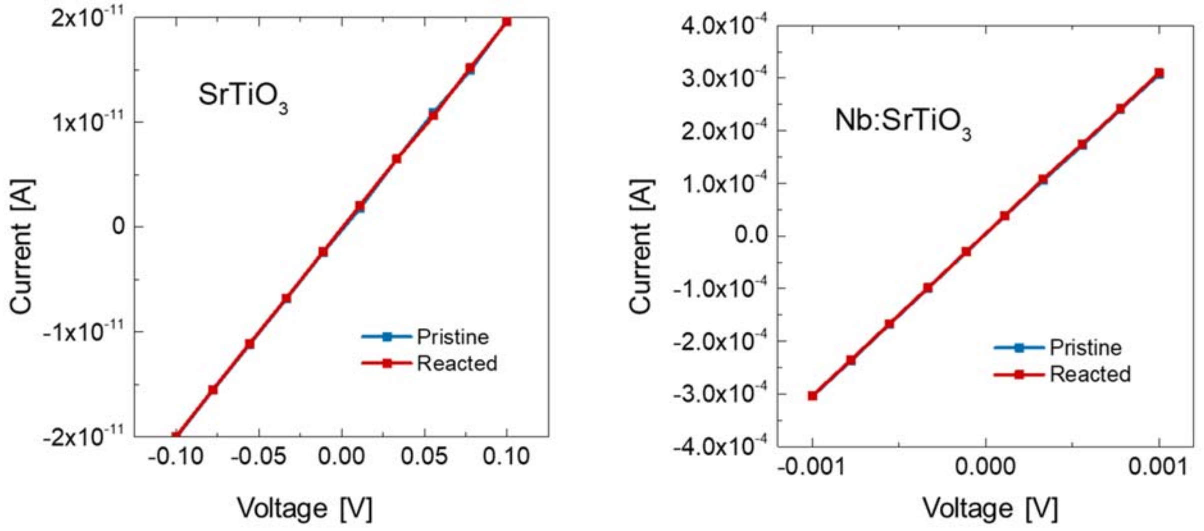

Supplementary Figure 7. Absence of reaction with GOx modified $SrTiO_3$ and Nb doped $SrTiO_3$. Perovskite oxides with empty *d* orbitals ($SrTiO_3$) and partially filled *d* orbitals (Nb: $SrTiO_3$, wt0.7%) were decorated with GOx enzyme similar to the nickelate devices and reacted with 0.5 M glucose solution for 30 minutes. No measurable change of the device resistance was observed in either case.

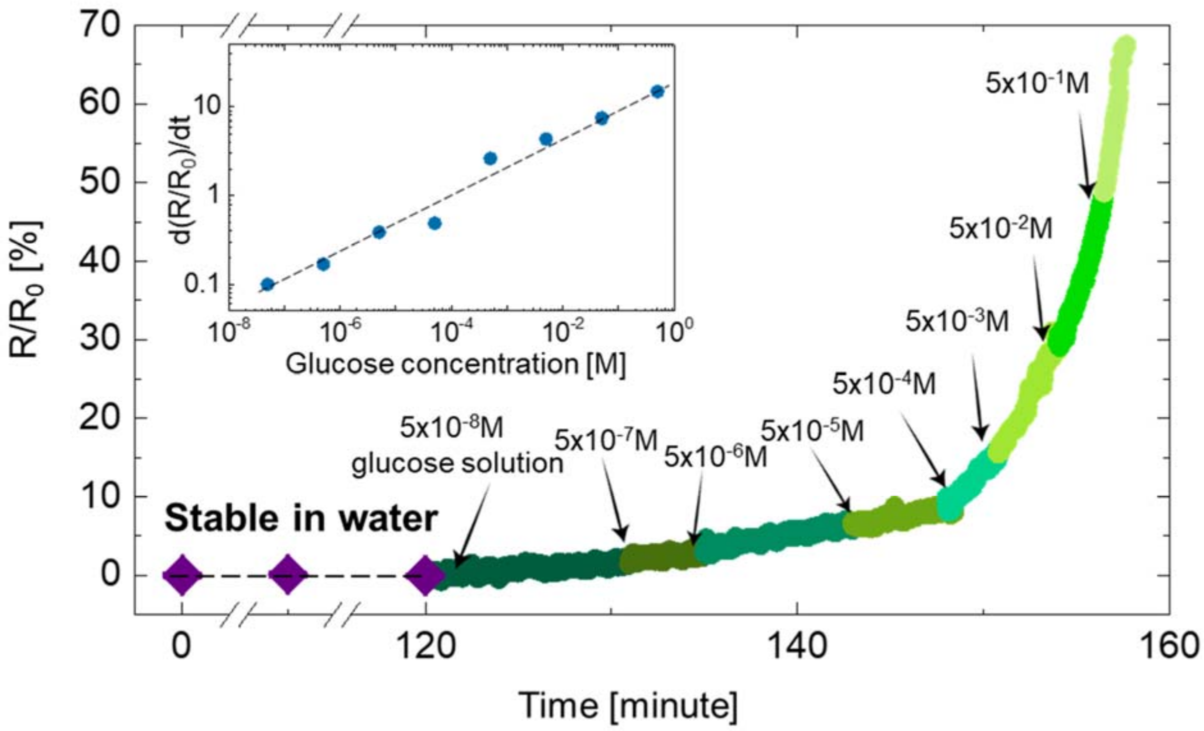

Supplementary Figure 8. Stability of the device in water and response to varying glucose concentration. The stability of the GOx-SNO device in water was tested by immersing this device in DI water and no resistance change was noticed, suggesting good stability of SNO. Then as glucose solution of different concentration was applied, the device became active and a clear resistance increase was observed due to the hydrogen transfer. A larger slope of resistance increase as a function of time $((\Delta R/R_0)/dt)$ was observed with higher glucose concentration, and the slope can be used as characteristic response to identify specific glucose concentration (shown in the inset). The dash lines are guides for the eye.

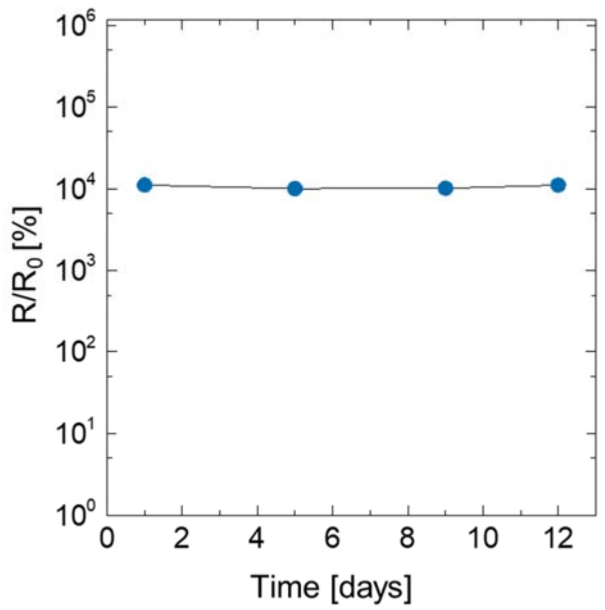

Supplementary Figure 9. Stability of the glucose reacted GOx-SNO device in ambient atmosphere. The resistance change of a GOx-SNO device was monitored over several days, after its reaction with 0.5 M glucose solution for 1 hour. The resistance change remained stable (non-volatile) and no degradation was found for the reacted device.

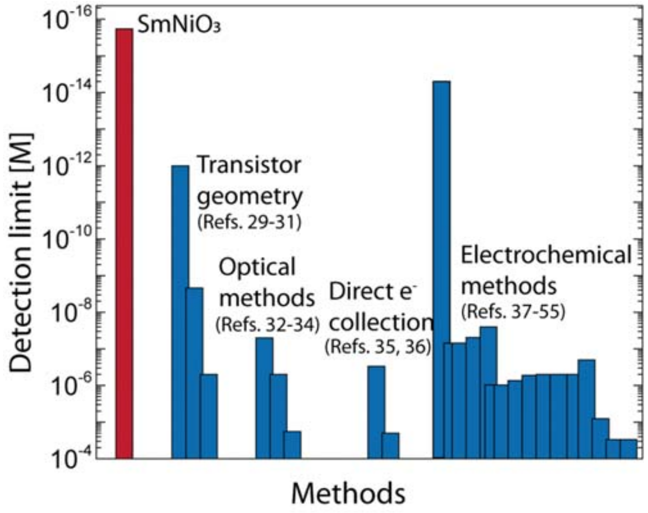

Supplementary Figure 10. Summary of glucose sensing mechanisms and detection limits. Literature reports (mainly focused on past ten years of literature representative of recent results) on glucose sensing mechanisms can be summarized into four categories: field effect transistor devices[5–7], optical methods[8–10], direct electron (e^-) collection[11,12], and electrochemical methods[13–31]. The field effect transistor devices utilize gate voltage signal from the glucose reaction to modulate the channel conductance for glucose detection. Optical methods were performed by monitoring the optical emission/properties change at certain wavelength that is specific to the glucose reaction. Direct electron collection measures the glucose concentration by directly collecting the electron signal in certain glucose reactions. Electrochemical methods involve cyclic voltammetry to detect the reduction/oxidation peaks of glucose. The glucose detection limit is typically between 10^{-6} to 10^{-8} M, with the highest value being 10^{-12} M for the transistor device,[5] and 2×10^{-15} M for the voltage based electrochemical method with a specially designed reaction cell.[18] The mechanism reported in this work is based on extreme electron localization in $SmNiO_3$ by carrier doping and has a detection limit of ~5×10^{-16} M.

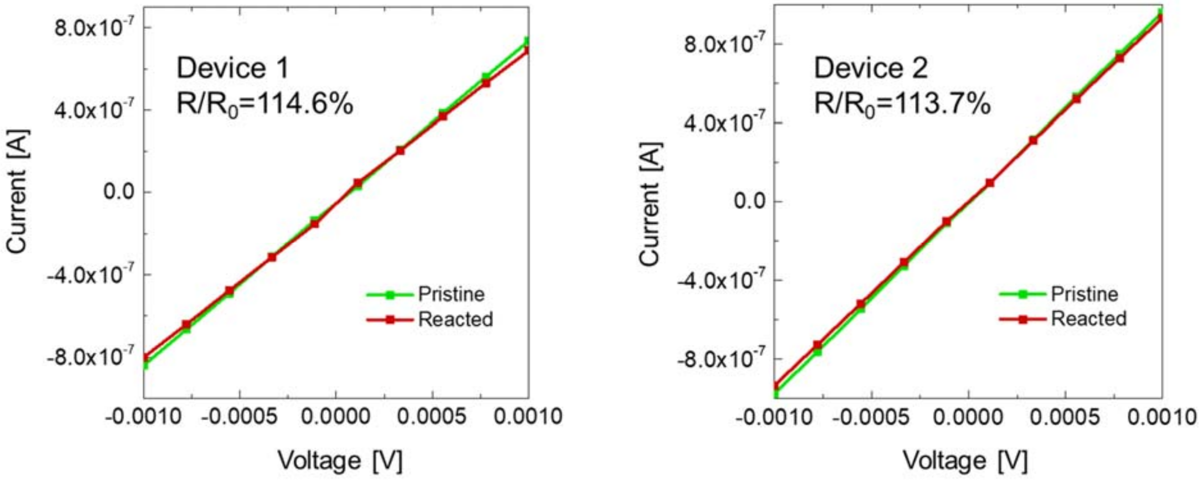

Supplementary Figure 11. Device reproducibility measurements. Two additional GOx-SNO devices were reacted with low concentration glucose solution ($5x10^{-16}$M) and increase of resistance ratio (R/R_0) was consistently observed, demonstrating reproducibility.

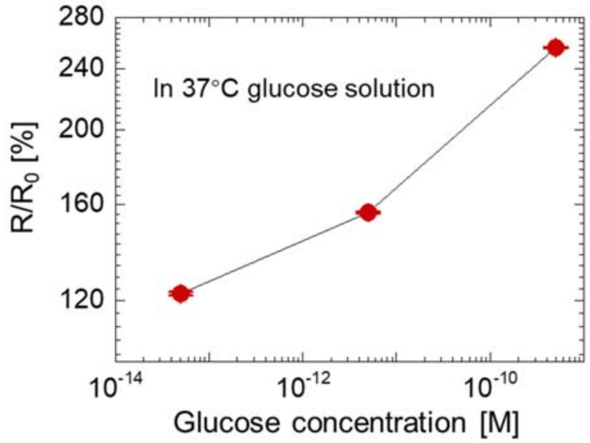

Supplementary Figure 12. Performance of the GOx-SNO device at body temperature (37 °C). The GOx-SNO device was soaked in glucose solution (of varying concentrations) at human body temperature (37 °C) for 1 hour and then its resistance was measured. The device is functional at body temperature and a systematic increase of resistance ratio (R/R_0) as a function of glucose concentration was observed similar to experimental observations at room temperature.

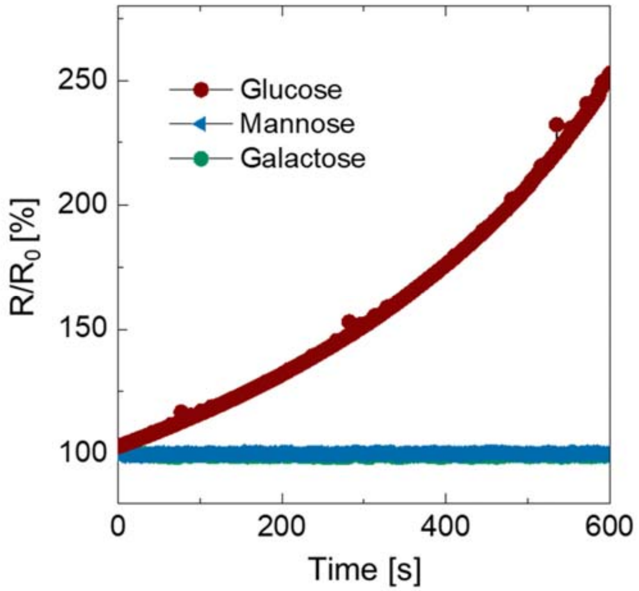

Supplementary Figure 13. Selectivity of the GOx-SNO device to glucose. To test the selectivity of the GOx-SNO device with different sugars other than glucose, reactions between the GOx-SNO device and glucose, mannose and galactose sugar solution was monitored by measuring the resistance of the device *in-situ*. The GOx-SNO device showed good selectivity and no resistance change was found for mannose and galactose solutions.

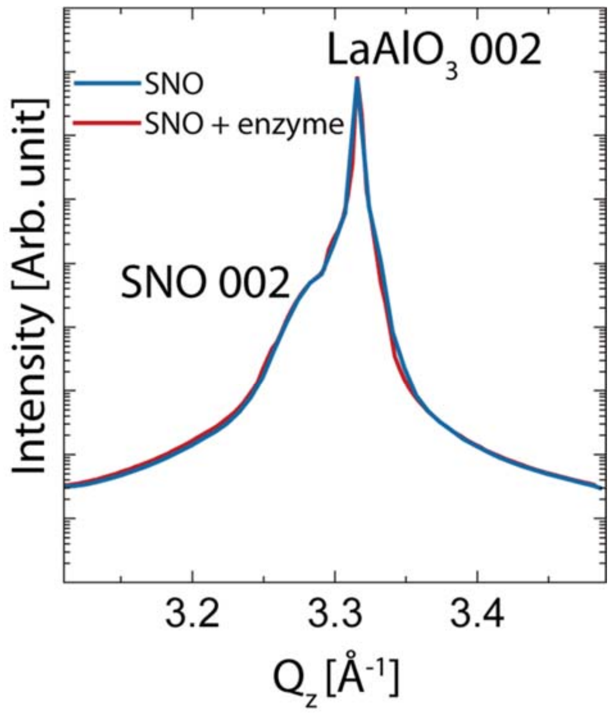

Supplementary Figure 14. X-ray diffraction (XRD) data of $SmNiO_3$ film. XRD data of the pristine SNO film before and after GOx enzyme attachment, along Q_z direction around the 002 peak (pseudocubic notation) of $LaAlO_3$ substrate. SNO (002) peak was observed with smaller Qz due to larger out of plane lattice constant of SNO compared to that of the $LaAlO_3$ substrate. After the GOx enzyme modification, no change was found in the XRD scan.

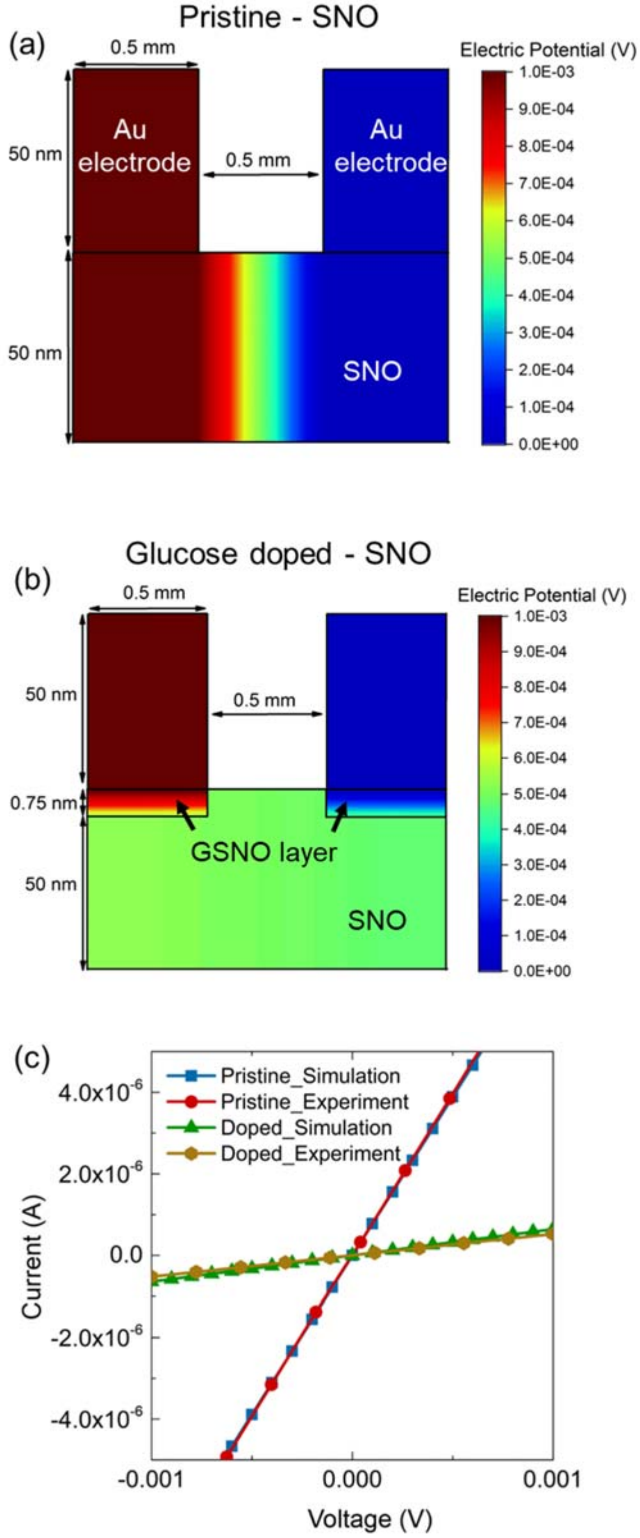

Supplementary Figure 15. COMSOL simulation of electron transport in SNO and GSNO. (a) Electric potential profile of simulated structure for SNO in pristine state shows that the entire potential drop is across the SNO region between the contacts. **(b)** Electric potential profile of simulated structure for glucose-doped SNO which shows that the potential drop is divided across the ultra-thin hydrogen doped layers (GSNO) under contacts. Resistance ratio of 11.8, similar to the experimental results, is observed for a doped layer thickness

of 0.75 nm. The dimensions of the experimental device structure are shown in the figures. **(c)** Simulated IV curves show close agreement with experimental ones for the given resistance ratio. The thickness obtained thus gives a lower bound on the hydrogen doped GSNO layer and complements the XANES measurements.

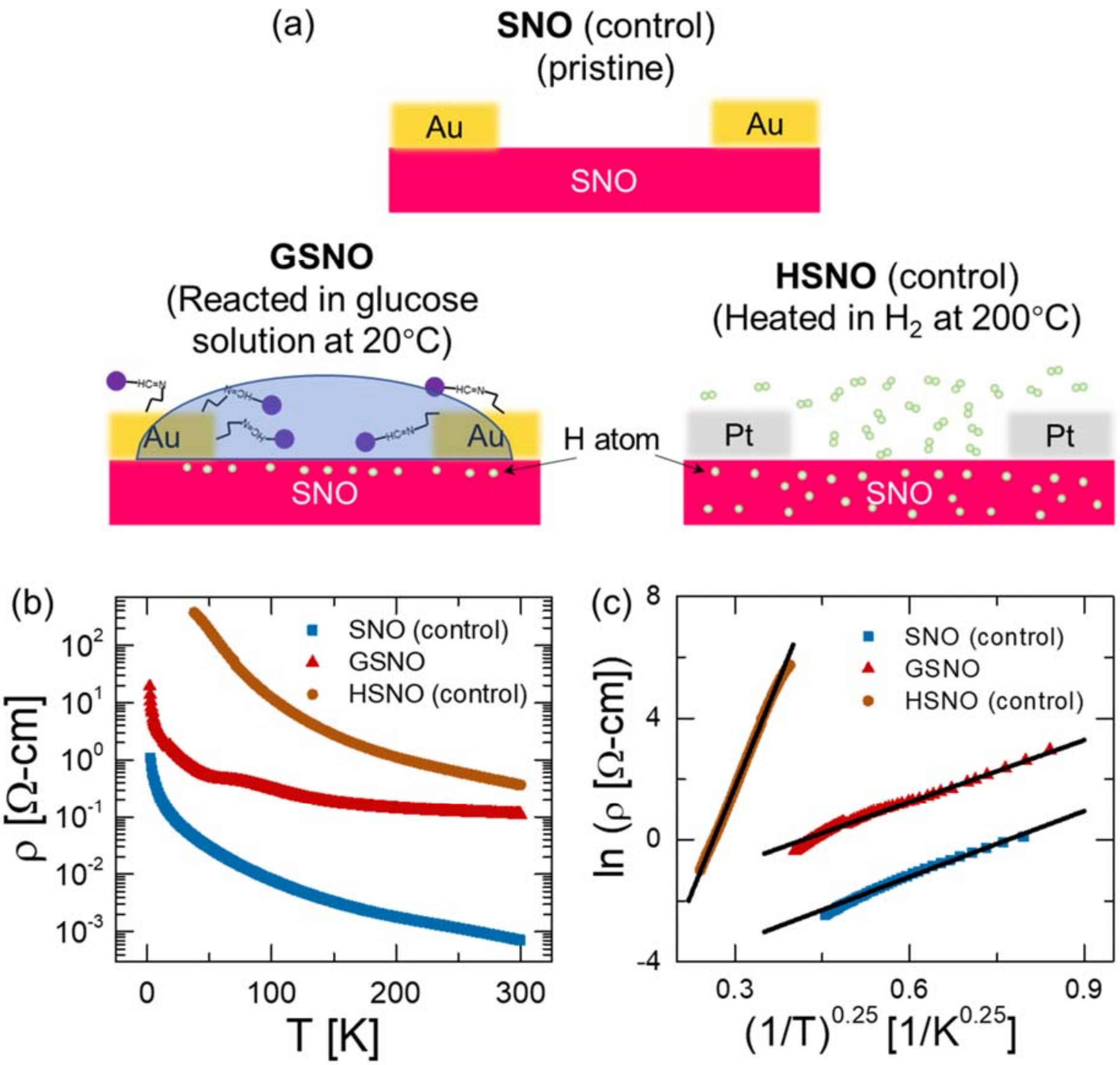

Supplementary Figure 16. Low temperature transport measurement of the SNO and GSNO samples. (a) Schematic figures of SNO under different treatment conditions: Pristine SNO sample, GSNO refers to SNO reacted with 0.5 M glucose solution for 1h, HSNO (control sample) refers to SNO that is heavily doped by annealing at 200 °C in gaseous H_2. **(b)** Temperature dependent resistivity of different samples: Pristine SNO, GSNO and HSNO control sample. The GSNO has resistivity in-between that of pristine SNO and HSNO, showing the effects of near-surface doping after its room temperature reaction with glucose solution. **(c)** Quantitative study on low temperature transport mechanism: ln (ρ) vs $T^{-1/4}$ was plotted for all samples and the black lines are fits for Mott variable range hopping.[32] Though GSNO displays higher resistivity than SNO, their slopes are similar, indicating that the carrier localization length is similar to that of pristine SNO (~37 nm). Although GSNO has a resistivity higher than the pristine SNO due to local doping near the electrode interface, the similar carrier localization length indicates the majority of the film is still SNO. The data of SNO and HSNO was taken from Ref. [32] for comparison purposes. The analysis further supports the synchrotron XANES experiments and COMSOL transport simulations indicating the nanoscale near-surface confinement of hydrogen doped from glucose that is kinetically limited due to the room temperature enzymatic reactions.

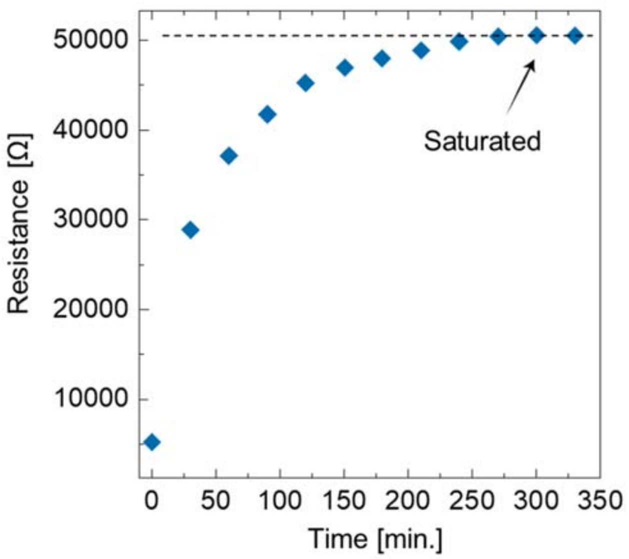

Supplementary Figure 17. Multiple use of the GOx-SNO device. The GOx-SNO device was soaked in 0.5M glucose solution and the device resistance was measured periodically. Eventually, when the top layer SNO was fully doped (due to self-limiting kinetics near room temperature), the device resistance would be saturated. The device can be used multiple times before the saturation point is reached since each sensing measurement can be accomplished in a few minutes.

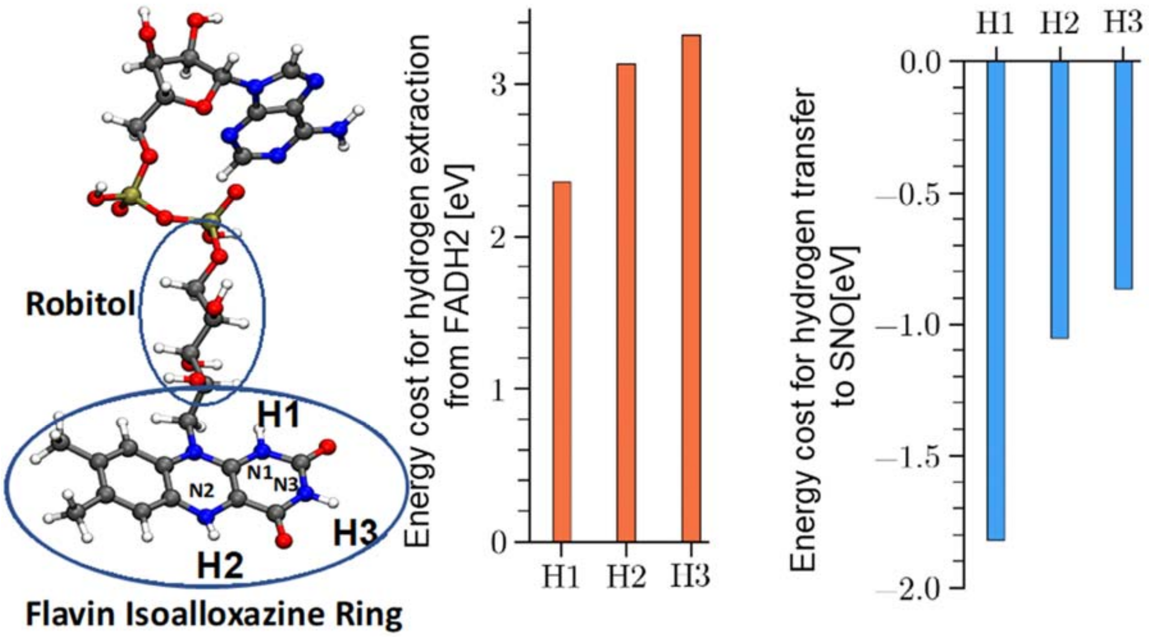

Supplementary Figure 18. Calculations showing spontaneous hydrogen transfer from FADH2 to SNO. Energetic cost to extract a hydrogen from FADH2 to SNO (001) computed using density functional theory (DFT) calculations (see Methods); three different active sites in FADH2 are considered and compared as shown in the schematic. H1, H2 and H3 represent possible sites for extraction. Energetic cost to dehydrogenate FADH2 follows the H3 > H2 > H1. With the presence of SNO, the transfer from FADH2 to SNO is energetically favored with a negative heat of formation. Note that H1 and H2 are transferred from glucose molecules and are energetically easier to extract.

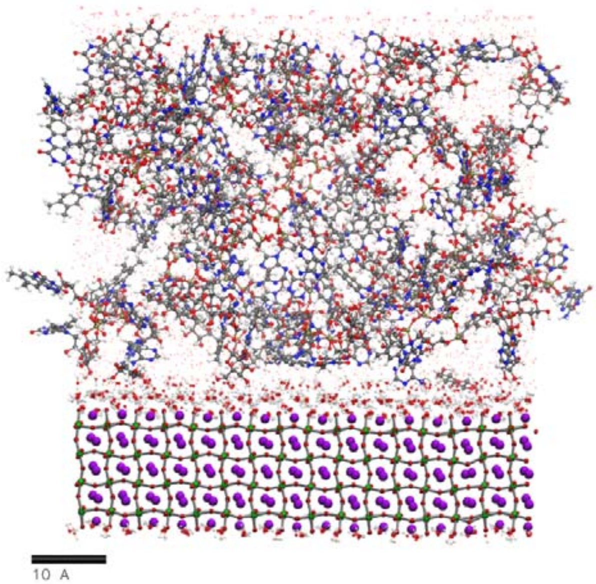

Supplementary Figure 19. Sampling initial configuration for the classical MD simulations. Molecular dynamics (MD) simulations based on empirical force fields are performed to study the conformational dynamics of gluconolactone and hydrogenated cofactor flavin adenine dinucleotide (FADH2) in water, in close proximity to a SNO slab. Snapshot shows representative interface between SNO (001) surface and gluconolactone/FADH2/water molecules. Different concentrations (from 0.01 M to 0.5 M) were prepared and simulated.

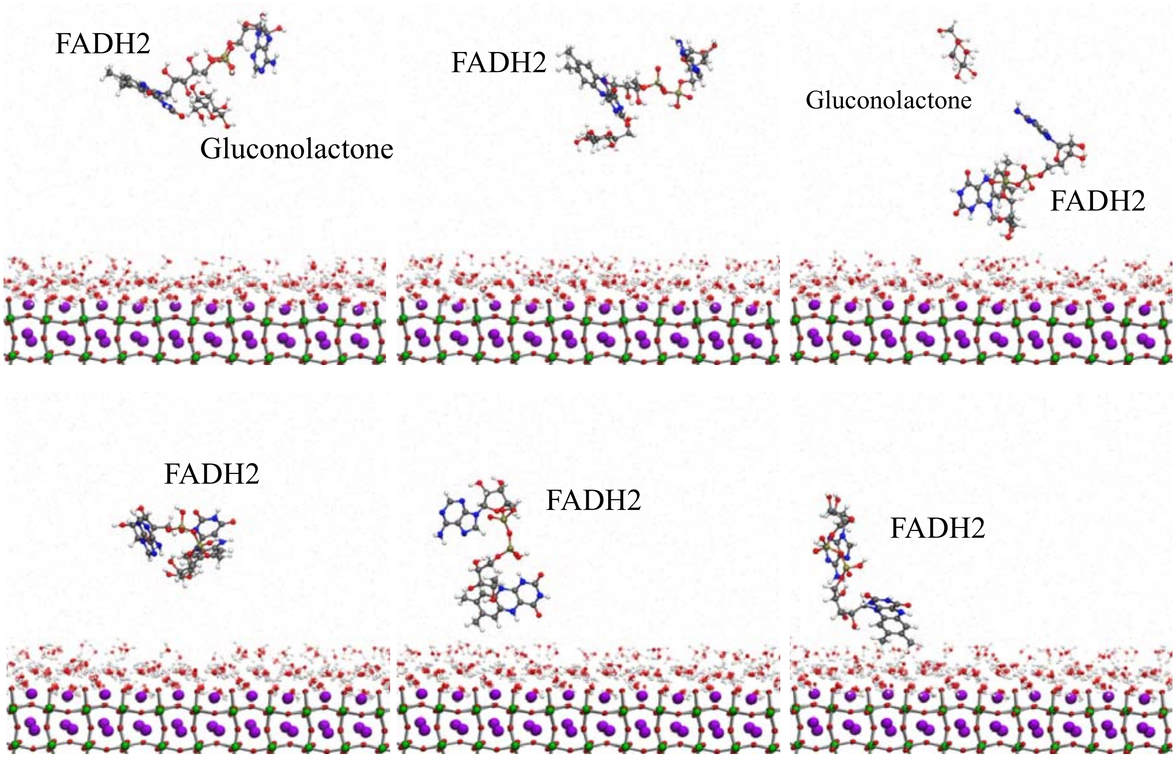

Supplementary Figure 20. Conformational dynamics during the approach of FADH2 molecule. The FADH2 molecules undergoes a series of conformational changes before adsorbing onto SNO (001) plane over 10 ns timescale. We sample several such energetically favorable near-surface configurations of FADH2 from the classical MD and use it as starting configurations for smaller AIMD models. Our AIMD simulations employ generalized gradient approximation (GGA), while using the projector-augmented wave formalism. The exchange correlation is described by the Perdew-Burke-Ernzerhof (PBE) functional,[33] with the pseudopotentials: Sm_3 (valence: $5s^2 5p^2 6s^2 4f^1$), Ni_pv (valence: $3p^6 4s^2 3d^8$) and O (valence $2s^2 2p^4$) supplied by VASP. These AIMD simulations are used to study the effects of strong correlation and its role in FADH2 dehydrogenation for several sterically favorable conformations (~30) sampled from classical MD.

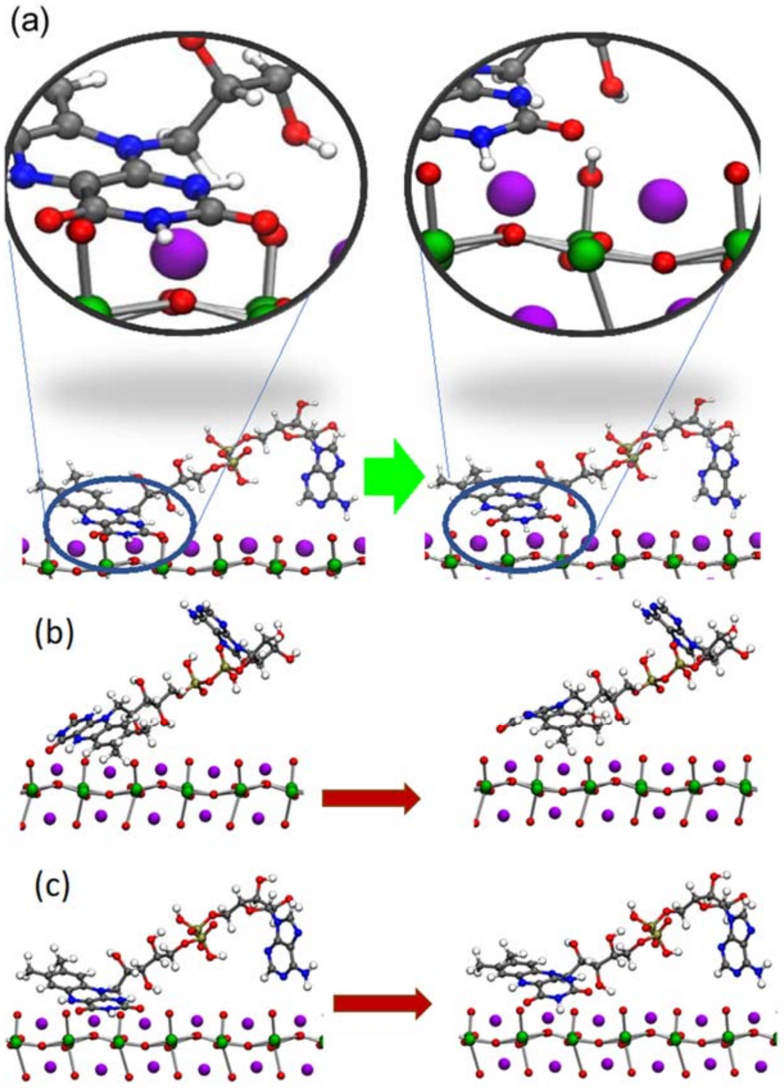

Supplementary Figure 21. AIMD simulation trajectories starting with different configurations. The conformation of the FADH2 sampled from the classical MD runs plays a key role in the hydrogen transfer. While some configurations display spontaneous hydrogen transfer as shown in (a), there are also cases where the steric effects hinder [(b) and (c)] the transfer within the AIMD timescales.

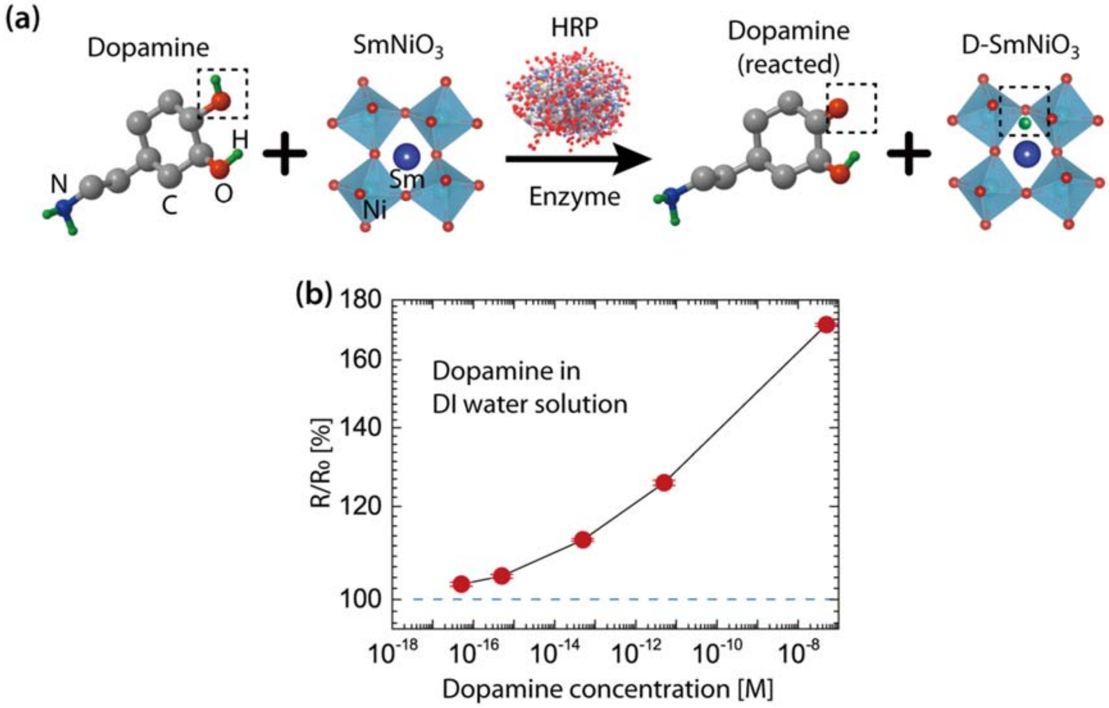

Supplementary Figure 22. Spontaneous hydrogen transfer from dopamine to HRP-SNO device. (a) Reaction mechanism between the SNO-HRP device and dopamine. In this reaction, HRP enzyme serves as a catalyst and transfers hydrogen from dopamine to SNO, referred to as D-SNO. The hydrogens bonded with carbons in the dopamine and HRP enzyme are omitted for figure clarity. **(b)** Electrical response of the HRP-SNO devices to varying dopamine concentration in DI water solution. The channel resistance change is presented as ratio before and after the reaction (R/R_0). The error bar was determined from the standard deviation of 10 measurements in each case.

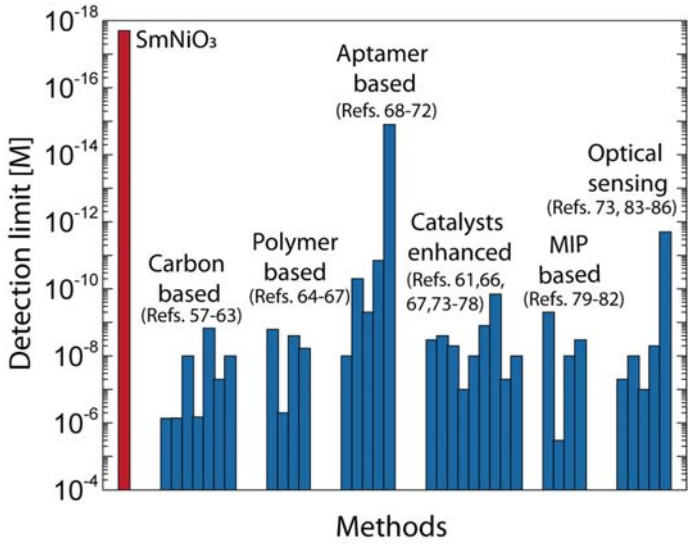

Supplementary Figure 23. Summary of dopamine sensing mechanisms and detection from literature. Representative literature reports on dopamine sensing mechanisms can be summarized into six categories: carbon-based methods,[34–40] polymer-based methods,[41–44] aptamer-based methods,[45–49] catalyst-enhanced methods,[38,43,44,50–55] molecular imprinted polymer (MIP) based methods[56–59] and optical sensing.[50,60–63] Carbon-based materials such as carbon nanotubes and graphene can be used to detect dopamine through electrical and electrochemical measurements. Polymer based sensors are used due to their biological compatibility. In the Aptamer based methods, short DNA or RNA oligomers are specially designed so that they bind with dopamine for its detection. Catalysts enhanced methods use catalysts to modify electrodes (such as polymer, graphene and carbon fiber etc.), and enhance sensor performance. In the molecular imprinted polymer (MIP) methods, functional monomers first polymerize with template molecules, and then the template molecules are removed. In this way, the detection surface area is increased, and the target molecules bind to the functional sites more preferentially. The optical sensing methods detect local change in optical properties or fluorescence due to the presence of dopamine. The mechanism reported in this work utilizes strong electron correlations induced by hydrogen doping leading to electron localization in $SmNiO_3$ and has a detection limit of $\sim 5\times10^{-17}$ M.

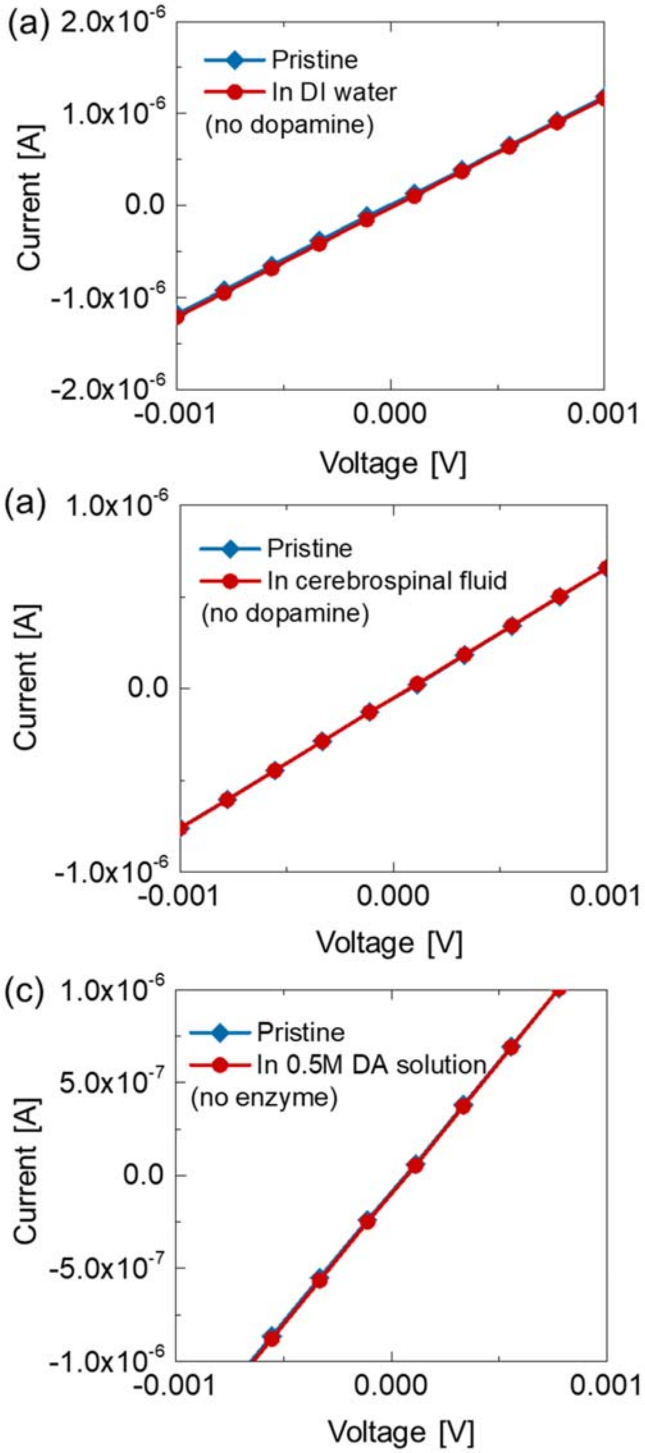

Supplementary Figure 24. Control experiments for the reaction with dopamine and HRP-SNO devices. **(a)** The HRP-SNO device was soaked in DI water for 1 hour and no resistance change was observed, indicating the HRP-SNO device is stable in DI water. **(b)** The HRP-SNO device was soaked in artificial cerebrospinal fluid (ACSF) for 1 hour and then the device resistance was measured. The device was found to be stable in ACSF and no degradation was found. **(c)** SNO device without HRP enzyme decoration was soaked in 0.5 M dopamine DI water solution for 1 hour and no resistance change was observed. This result indicates that the HRP enzyme is necessary for hydrogen transfer from dopamine to SNO.

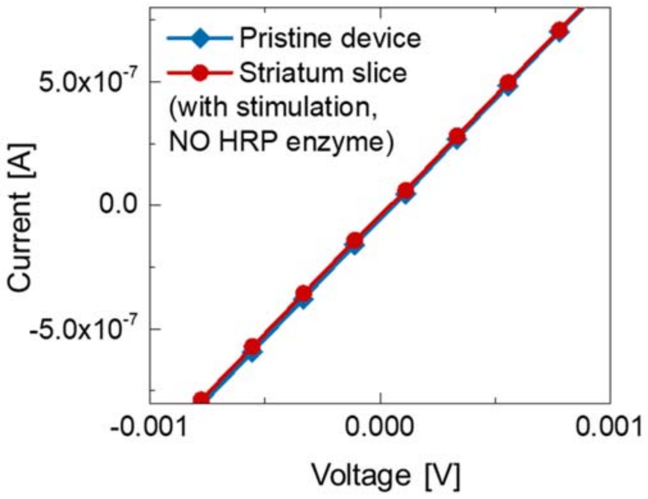

Supplementary Figure 25. Control for the mouse brain interfacing experiment without HRP enzyme. When the stimulated striatum slice is interfaced with SNO film *without* any HRP enzyme, negligible resistance change was observed, indicating the enzyme assisted hydrogen transfer is the principal mechanism operational in HRP-SNO devices.

Supplementary Tables

Supplementary Table 1. Summary of all samples used in this work and their labels

Sample	Treatment	Symbol	Manuscript	Supplement
Pristine $SmNiO_3$	None	SNO	Figure 3	Figure S2, S3, S4, S14, S16, S24, S25
$SmNiO_3$ modified by GOx enzyme	Reaction with glucose solution in DI water at room temperature.	GSNO	Figure 2, 3	Figure S5, S8, S9, S11, S13, S16
$SmNiO_3$ modified by HRP enzyme	Reaction with dopamine solution in DI water at room temperature.	DSNO	Figure 4	Figure S21, S24, S25
$SmNiO_3$ fully doped by hydrogen by annealing in H_2 gas (control)	Annealed in forming gas (95% N_2/5% H_2) at 200 °C.	HSNO	None	Figure S16

Supplementary Methods

$SmNiO_3$ sample growth and device fabrication

50 nm thick $SmNiO_3$ (SNO) films were grown using an AJA UHV sputtering system. Before deposition, $LaAlO_3$ substrates were rinsed by acetone and isopropanol, after which the substrates were dried by nitrogen. Co-sputtering of Sm and Ni targets were performed with Sm at a radio frequency (RF) power at 170W, and Ni at direct current (DC) power at 70W. This growth condition ensures a stoichiometric ratio of Sm to Ni cations as characterized by energy-dispersive X-ray spectroscopy (EDS). The growth was carried out at room temperature with a background pressure of argon and oxygen mixture at 5 mTorr. Then the film was annealed at 500 °C for 24 hours under 100 bar of O_2 in a home-built high-pressure tube furnace. Au electrodes (50 nm thick with 3nm Ti adhesion layer) were deposited using electron beam evaporation, and the devices were fabricated with shadow masks (500 μm gap).

GOx-SNO and HRP-SNO enzyme anchored device fabrication

The GOx enzyme is selective to glucose oxidation while the horseradish enzyme (HRP) can be used to study dopamine release. To anchor the GOx enzyme and HRP enzyme respectively to the Au electrode surface,[64,65] the SNO devices were first immersed in 10 mM cystamine solution for 2 hours at room temperature in dark. The GOx enzyme (Sigma-Aldrich catalog number G6125), HRP enzyme (Sigma-Aldrich catalog number P8375) and cystamine were purchased from Sigma-Aldrich Corporation. The SNO devices were then rinsed with DI water to remove the unreacted cystamine and dried with compressed air. Next, the glucose oxidase (GOx) was oxidized in order to conjugate to the cystamine on the Au surface. For this purpose, 30 mg sodium metaperiodate was added into 20 μM GOx solution (in 5 mL 0.1 M pH 6.8 Sodium Phosphate buffer). The mixture was incubated in 4 °C for 1 hour. Then 6.97 μL ethylene glycol was added into the mixture and incubated at room temperature for 30 mins to stop the reaction. This product was purified by PD-10 desalting column (GE Healthcare) to collect oxidized GOx as well as changing the buffer back to 0.1 M pH 6.8 Sodium Phosphate. The reacted SNO device was immersed in the collected GOx solution for 1 hour at 4 °C and then rinsed with DI H_2O. The device was then dried by compressed air and stored at 4 °C ready for use. This process is schematically illustrated in **Figure S1** using GOx as an example. The mannose and galactose used in the selectivity measurement were also purchased from Sigma-Aldrich Corporation.

Atomic force microscopy

The morphology of the SNO devices with GOx decorated Au surface and bare Au surface were characterized with a Bruker Dimension Icon atomic force microscope under the PeakForce Quantitative Nanomechanical Mapping in Air mode.

Electrical and electrochemical measurements

For the *in-situ* resistance characterization of the reaction between GOx-SNO devices and glucose solution, 20 μL of glucose solution was added on top of the SNO device and the device resistance was monitored using a Keithley 2635A source meter. To avoid any side-reactions, the *in-situ* measurement was performed in a chamber with constant Ar gas flow. Before reaction, the glucose solution was purged for 10 minutes with nitrogen gas. For *ex-situ* reaction characterization, the GOx-SNO and HRP-SNO devices were soaked in glucose and dopamine solutions for various times as mentioned in the manuscript, and then the resistance was measured with the Keithley 2635A

source meter. In both cases, the sweeping bias ranged from -0.001 to 0.001 V. Low temperature electronic transport measurements (from 2 K to 300 K) were performed in a PPMS system.

The cyclic voltammetry measurements to confirm the existence of GOx on top of the Au surface was performed with a Solartron 1260A electrochemical analyzer in a three-terminal configuration, with the Au surface as working electrode, a graphite rod as counter electrode, and Ag/AgCl (saturated KCl) as reference electrode. The electrolyte was 0.1 M pH 6.8 Sodium Phosphate buffer. For the CV measurement, the Au electrodes were deposited on top of a Si substrate to avoid any electrochemical reactions that involves the SNO.

Fourier-transform infrared spectroscopy

Fourier-transform infrared (FTIR) spectroscopy was performed with a Nicolet Nexus 470 FTIR in the absorption mode, which is computer-controlled via a OMNIC software. The Smart-iTR setup was used with a versatile Attenuated Total Reflectance (ATR) sampling accessory. The scan resolution is 4 cm^{-1} and the signal was accumulated over 36 scans.

Synchrotron X-ray diffraction and X-ray absorption spectroscopy

To characterize lattice structure and chemical state information at local regions in the reacted enzyme-anchored SNO devices, both X-ray diffraction (XRD) and X-ray absorption spectroscopy (XAS) measurements were carried out at the undulator beamline sector 33-ID-D of the Advanced Photon Source (APS) in Argonne National Laboratory. The devices were mounted on a Kappa-geometry six-circle diffractometer. To obtain local structural and chemical state information across the patterned region of the SNO devices, a focused X-ray beam with the profile of 80 μm (horizontal) × 18 μm (vertical) by an achromatic Kirkpatrick-Baez focusing X-ray mirror was used to collect the XRD pattern and XAS spectra from each selected spot. The XRD measurements were conducted at X-ray energy of 20 keV using a pixel array area detector (Dectris Pilatus 100 K). The X-ray beam has a flux of 2×10^{12} photons/second at 20 keV. The q_z-scan (L-scan) was acquired by removing the background signals using the two-dimensional detector images. The Ni K-edge XAS were performed at 33-ID-D using linear polarized X-rays from its Si (111) monochromator with energy resolution $\delta E/E = 1 \times 10^{-4}$. The X-ray beam has a flux of 6×10^{12} photons/second at around 8.33 keV. The XAS spectra were collected in the fluorescence mode using a Vortex 4-element silicon drift detector. A high purity Ni metal foil was used as a standard reference of the monochromator energy calibration. To obtain surface-sensitive XAS spectra of the patterned SNO samples, grazing incidence geometry was adopted to enhance the varied surface layer signal as well as minimize the elastic scattering background. The angle of X-ray incidence is tuned from 0.05° to 5.05°, spanning a wide range below and above the substrate-defined critical angle. At 0.05° angle of incidence, we can observe the most pronounced changes in the near-edge XAS spectra. All XRD and XAS measurements were performed at room temperature in ambient pressure. XAS spectra were processed and analyzed by using the Athena program (https://bruceravel.github.io/demeter/).

COMSOL simulations

Representative devices used in the experiments were simulated in COMSOL Multiphysics considering a thin hydrogen doped SNO layer (H-SNO) under the Au contacts in order to estimate the lower bound of the thickness of the H-SNO layer. This length scale complements the dopant depth scale inferred from variable angle X-ray absorption spectroscopy (upper bound). The H-SNO layer below the gold contact essentially forms an ultra-thin insulator sandwiched between two metallic layers. The large band gap (E_g) of H-SNO (~3eV) leads to tunneling transport.[3] To model the

tunneling resistance of H-SNO, we assume a direct tunneling phenomenon for asymmetrical barriers. We consider a linear resistance model where the resistivity of the layer is a function of thickness. Thus, resistance is $R(d) = \frac{V}{J(d)\times A} = \frac{\rho d}{A}$, and resistivity is given by $\rho(d) = \frac{V}{J(d)\times d}$. Note that due to the high aspect-ratio of the structure, an effective resistance method was adopted, where the H-SNO thicknesses was ten times higher than the concerned value and then the calculated resistance was divided by ten. At low voltages, the tunneling current follows an Ohmic regime and the current density is expressed as:[66]

$$J \approx \frac{e^2}{h}\left(\frac{1}{2\pi d}\right)\frac{\sqrt{2m\bar{\phi}}}{\hbar}\exp\left[-2\frac{\sqrt{2m\bar{\phi}}}{\hbar}d\right]V \qquad 1$$

where $\bar{\phi} = \frac{\phi_1+\phi_2}{2}$, ϕ_1 is the barrier height Au-H-SNO interface, ϕ_2 is the barrier-height of H-SNO-SNO interface, d is the thickness of the H-SNO layer, V is the applied voltage, $m\ (= 7m_0)$ is the effective mass of H-SNO (we assume here it is the same value as that of un-doped SNO),[67] h is Planck's constant, e is electron charge. The barrier heights are calculated as $\phi = WF - \chi$. Work-function of Au and SNO are 5.1 eV[68] and 4.93 eV[69] respectively and electron affinity χ of SNO is 3.43 eV ($WF_{\mathrm{SNO}} - E_{\mathrm{g}}/2$).

Hybrid computational approach to understand biomolecule interaction with quantum material

Hybrid approaches such as QM/MM have been used to bridge typical time and length scales, and enhanced sampling simulation techniques to accelerate adsorption and desorption events have been proposed and successfully applied to study biomolecule–inorganic surface interactions. In the past, biomolecules such as proteins on surfaces have been simulated predominantly using static DFT calculations of single amino acids or even di- and tri-peptides.[70,71] Other examples of these kind of calculations are works on Cys–Au(111) interactions,[72–74] often used for protein immobilization. DFT calculations, in the past, have also been used to elucidate amino acid adsorption on other inorganic surfaces such as silica,[75] hydroxyapatite,[76] alumina[77] and titania surfaces.[78] While quantum simulations have been carried out extensively for interaction of biomolecules with metal as well as even oxide surfaces, where electrostatic interactions and hydrogen bonding as well as chemical bonding mechanisms are important, studies on interaction of biomolecules with strongly correlated oxides are scarce. To the best of our knowledge, the hybrid approach (MD/AIMD) presented here represents the first attempt to understand the impact of strong correlations and electronic effects arising from a quantum material on biomolecules such as hydrogenated cofactor flavin adenine dinucleotide (FADH2).

Classical molecular dynamics simulations

Molecular dynamics (MD) simulations based on empirical force fields are performed to study the dynamics of gluconolactone and FADH2 in water, near a SNO slab. An interface between SNO (001) plane (pseudocubic notation) and glucose/FADH2/water molecules of different concentrations (from 0.01 M to 0.5 M) are prepared and simulated. These molecules are randomly orientated and initially placed at least 0.5 nm away from the surface of the SNO slab inside a 6 nm × 6 nm × 8 nm periodic simulation box that is filled with explicit water molecules (system size up to 30k atoms). The Cartesian coordinates of the FADH2 is initially taken from the protein data bank

(https://www.rcsb.org/). This cofactor structure is minimized using Gaussian 09 at HF/6-31G(d,p). Configuration of the monoclinic SNO slab (5120 atoms) is created from a DFT energy minimized structure, and the stability of the slab is maintained by fixing the position of (Ni, Sm, O) atoms within the slab throughout the simulations. Partial charges of 1.4, 3.4, and -1.6 are respectively assigned to Ni, Sm, and O atoms within the SNO slab, which are based on Bader charges from DFT calculations while keeping the sum of charges neutral. The intra- and inter- molecular interactions between all atoms are modeled using the CHARMM force field,[79–81] which describes bonded interactions (bonds, angles, dihedrals, improper) within molecules using harmonic type potentials (i.e., fixed bond) and non-bonded interactions using standard Coulomb electrostatics and Lenard-Jones potentials. A 1 nm cutoff is used for the non-bonded interactions and a Particle mesh Ewald algorithm is used to capture the long-range electrostatics. Water molecules are modeled by rigid 3-site (TIP3P) model to ensure computational efficiency and compatibility with the chosen force field. All classical MD simulations in this study are performed using the Nanoscale Molecular Dynamics (NAMD) simulator using a simulation timestep of 2 fs. Since atoms in the SNO slab are spatially fixed, isothermal-isobaric (NPT) ensemble is employed with the in-plane x- and y- dimensions fixed, i.e., only the z- dimension (perpendicular to the SNO slab) is allowed to change. Thermostat and barostat based on Langevin dynamics are employed to maintain the system temperature at 300K and pressure at 1 bar. A damping coefficient of 0.01 ps^{-1} is used for the thermostat while a 0.2 ps oscillation period and a 0.1 ps decay time is used for the barostat.

Ab initio molecular dynamics simulations

To understand the interaction between Flavin adenine dinucleotide (FADH2) and exposed surface of SNO, we perform *ab initio* molecular dynamics (AIMD) simulations on sterically favorable configurations sampled from the classical MD simulations. The size of the systems ~ 400 atoms coupled with the presence of a quantum material such as SNO make the AIMD calculations computationally intensive. As such, these simulations required us to use the leadership computing facilities at Argonne National Lab (~2048 to 8192 cores for each run).[78]

All the AIMD simulations are carried out within the generalized gradient approximation (GGA), while using the projector-augmented wave formalism as implemented in the Vienna Ab-initio Simulation Package (VASP).[82,83] The exchange correlation is described by the Perdew-Burke-Ernzerhof (PBE) functional,[33] with the pseudopotentials: Sm_3 (valence: $5s^2 5p^2 6s^2 4f^1$), Ni_pv (valence: $3p^6 4s^2 3d^8$) and O (valence $2s^2 2p^4$) supplied by VASP. The plane wave energy cutoff is set at 520 eV and the Brillouin zone was sampled at Γ-point only. The computational supercell is composed of two unit cells of SNO (001) slab (320 atoms). The bottom layers containing 80 atoms are fixed to improve computational efficiency. Periodic boundary conditions are employed along all directions with a vacuum of ~10 Å along the surface normal. Using isobaric-isothermal (NPT) ensemble, we first thermalize the SNO computational supercell at 300 K and zero external pressure for 10 ps using a time step of 0.5 fs. The temperature conditions are maintained by using a Langevin thermostat, while the atomic positions, supercell shape and volume are equilibrated using the Parinello-Rahman scheme.[84] Next, we introduce one molecule of FADH2 at different locations with arbitrary orientation above the SNO surface; we ensure that the periodic images along the surface normal are at least 10 Å apart to avoid spurious interactions. The FADH2-SNO systems are equilibrated at 300 K in the canonical ensemble (constant volume, temperature). Constant temperature conditions were maintained via Nose Hoover thermostat[85] as implemented in VASP.

Electronic structure calculations:

We assess the energetic feasibility of hydrogen abstraction from FADH2 to SNO surface by using density functional theory (DFT) calculations within the GGA+U framework (as described in Sec. S10). The exchange correlation functionals, energy cut-off, and Brillouin zone sampling are identical to those employed for AIMD simulations. The computational supercell consisted of a FADH2 molecule placed near the exposed surface of SNO (001) [2 unit cells; 320 atoms]; the shortest distance between FADH2 and SNO (001) is chosen to be ~2.5 Å. We have amply sampled adsorption locations as well as orientatations of FADH2 molecule relative to the exposed SNO surface (~30 configurations). For each of these starting configurations, we perform structural optimization *via* conjugate gradient relaxations until the atomic forces are less than 0.01 eV/ Å.

Acute mouse brain striatal and primary visual cortical slices preparation

All animal procedures were approved by Purdue Animal Care and Use Committee. Acute striatal and primary visual cortical slices were prepared as noted in literature.[86] C57/BL6 mice age between P31 and P33 were anesthetized with ketamine and xylazine mix (ketamine 100mg/ml, xylazine 16mg/ml in saline) at 1ml per 100g body weight. Deep anesthesia was confirmed by the absence of the toe pinch reflex. The animal was subjected to trans-cardiac perfusion with High Sucrose Dissection Buffer (HSDB) containing (in mM): 75 sucrose, 10 glucose, 87 NaCl, 2.5 KCl, 1.25 NaH_2PO_4, 25 $NaHCO_3$, 0.5 $CaCl_2$, 7 $MgCl_2$, 1.3 ascorbic acid. Then the brain was dissected and the part of the brain containing striatum, or the primary visual cortex was cut into 300µm coronal sections using a vibrating microtome (Leica VT1000) in oxygenated HSDB. Brain slices were transferred into oxygenated artificial cerebrospinal fluid (ACSF) which comprises (in mM) 124 NaCl, 3.5 KCl, 1 $CaCl_2$, 0.8 $MgCl_2$, 1.23 NaH_2PO_4, 26 $NaHCO_3$, and 10 glucose. The slices were then incubated at 32°C for 30min, and then at room temperature for at least 1h before the experiment. Slices were kept in ACSF for up to 6h.

Brain slice electrical stimulation protocol

Repeated high-frequency pulse trains were applied to trigger dopamine release in the striatum. Identical stimulation protocol was applied in control experiments. Electrical stimulation was generated by a stimulation isolator (WPI A365) and delivered through a bipolar electrical stimulating electrode (FHC CE2C55) on the surface of the brain slices in the region of interest (striatum in experimental condition and primary visual cortex in negative control). 20 pulses (5ms width, 350µA) at 20Hz consist a single pulse train, and one pulse train was applied every 15 seconds for a total of 120 times.

Supplementary References

1. Tajmir-Riahi, H.-A. Carbohydrate Complexes with Lead(II) Ion. Interaction of Pb(II) with β-D-Glucurono-6,3-lactone, D-Glucono-1,5-lactone, and Their Acid Anions and the Effects of Metal Ion Binding on the Sugar Hydrolysis. *Bull. Chem. Soc. Jpn.* **62,** 1281–1286 (1989).
2. Polshin, E. *et al.* Integration of microfluidics and FT-IR microscopy for label-free study of enzyme kinetics. *Sensors Actuators, B Chem.* **196,** 175–182 (2014).
3. Shi, J., Zhou, Y. & Ramanathan, S. Colossal resistance switching and band gap modulation in a perovskite nickelate by electron doping. *Nat. Commun.* **5,** 4860 (2014).
4. Zuo, F. *et al.* Habituation based synaptic plasticity and organismic learning in a quantum perovskite. *Nat. Commun.* **8,** 240 (2017).
5. Cella, L. N., Chen, W., Myung, N. V. & Mulchandani, A. Single-walled carbon nanotube-based chemiresistive affinity biosensors for small molecules: Ultrasensitive glucose detection. *J. Am. Chem. Soc.* **132,** 5024–5026 (2010).
6. Zhang, M. *et al.* Highly sensitive glucose sensors based on enzyme-modified whole-graphene solution-gated transistors. *Sci. Rep.* **5,** 8311 (2015).
7. Tang, H., Yan, F., Lin, P., Xu, J. & Chan, H. L. W. Highly sensitive glucose biosensors based on organic electrochemical transistors using platinum gate electrodes modified with enzyme and nanomaterials. *Adv. Funct. Mater.* **21,** 2264–2272 (2011).
8. Bo, T. *et al.* A New Nanobiosensor for Glucose with High Sensitivity and Selectivity in Serum Based on Fluorescence Resonance Energy Transfer (FRET) between CdTe Quantum Dots and Au Nanoparticles. *Chem. Eur. J.* **14,** 3637–3644 (2008).
9. Tian, J. *et al.* Ultrathin graphitic carbon nitride nanosheets: a novel peroxidase mimetic, Fe doping-mediated catalytic performance enhancement and application to rapid, highly sensitive optical detection of glucose. *Nanoscale* **5,** 11604–11609 (2013).
10. Jv, Y., Li, B. & Cao, R. Positively-charged gold nanoparticles as peroxidiase mimic and their application in hydrogen peroxide and glucose detection. *Chem. Commun.* **46,** 8017 (2010).
11. Wang, Z., Liu, S., Wu, P. & Cai, C. Detection of Glucose Based on Direct Electron Transfer Reaction of Glucose Oxidase Immobilized on Highly Ordered Polyaniline Nanotubes. *Anal. Chem.* **81,** 1638–1645 (2009).
12. Kang, X. *et al.* Glucose Oxidase–graphene–chitosan modified electrode for direct electrochemistry and glucose sensing. *Biosens. Bioelectron.* **25,** 901–905 (2009).
13. Niu, X., Lan, M., Zhao, H. & Chen, C. Highly sensitive and selective nonenzymatic detection of glucose using three-dimensional porous nickel nanostructures. *Anal. Chem.* **85,** 3561–3569 (2013).
14. Zhuang, Z. *et al.* An improved sensitivity non-enzymatic glucose sensor based on a CuO nanowire modified Cu electrode. *Analyst* **133,** 126–132 (2008).
15. Dong, Y. *et al.* Graphene oxide-Fe3O4 magnetic nanocomposites with peroxidase-like activity for colorimetric detection of glucose. *Nanoscale* **4,** 3969–3976 (2012).
16. Ding, Y. *et al.* Electrospun Co3O4 nanofibers for sensitive and selective glucose detection. *Biosens. Bioelectron.* **26,** 542–548 (2010).
17. Dong, X. C. *et al.* 3D graphene-cobalt oxide electrode for high-performance supercapacitor and enzymeless glucose detection. *ACS Nano* **6,** 3206–3213 (2012).
18. Mano, N. & Heller, A. Detection of glucose at 2 fM concentration. *Anal. Chem.* **77,** 729–732 (2005).

19. Wang, Y. *et al.* Perovskite LaTiO3-Ag0.2 nanomaterials for nonenzymatic glucose sensor with high performance. *Biosens. Bioelectron.* **48,** 56–60 (2013).
20. Wang, L. *et al.* Perovskite-type calcium titanate nanoparticles as novel matrix for designing sensitive electrochemical biosensing. *Biosens. Bioelectron.* **96,** 220–226 (2017).
21. He, J. *et al.* High-performance non-enzymatic perovskite sensor for hydrogen peroxide and glucose electrochemical detection. *Sensors Actuators, B Chem.* **244,** 482–491 (2017).
22. Dai, H. *et al.* Synthesis of perovskite-type SrTiO3nanoparticles for sensitive electrochemical biosensing applications. *J. Electroanal. Chem.* **810,** 95–99 (2018).
23. Sivakumar, M., Pandi, K., Chen, S.-M., Cheng, Y.-H. & Sakthivel, M. Facile synthesis of perovskite-type NdNiO3 nanoparticles for an effective electrochemical non-enzymatic glucose biosensor. *New J. Chem.* **41,** 11201–11207 (2017).
24. Ci, S. *et al.* Nickel oxide hollow microsphere for non-enzyme glucose detection. *Biosens. Bioelectron.* **54,** 251–257 (2014).
25. Wang, J. & Zhang, W.-D. Fabrication of CuO nanoplatelets for highly sensitive enzyme-free determination of glucose. *Electrochim. Acta* **56,** 7510–7516 (2011).
26. Nie, H., Yao, Z., Zhou, X., Yang, Z. & Huang, S. Nonenzymatic electrochemical detection of glucose using well-distributed nickel nanoparticles on straight multi-walled carbon nanotubes. *Biosens. Bioelectron.* **30,** 28–34 (2011).
27. Luo, J., Jiang, S., Zhang, H., Jiang, J. & Liu, X. A novel non-enzymatic glucose sensor based on Cu nanoparticle modified graphene sheets electrode. *Anal. Chim. Acta* **709,** 47–53 (2012).
28. Jiang, L.-C. & Zhang, W.-D. A highly sensitive nonenzymatic glucose sensor based on CuO nanoparticles-modified carbon nanotube electrode. *Biosens. Bioelectron.* **25,** 1402–1407 (2010).
29. Bai, Y., Sun, Y. & Sun, C. Pt–Pb nanowire array electrode for enzyme-free glucose detection. *Biosens. Bioelectron.* **24,** 579–585 (2008).
30. Wei, H. & Wang, E. Fe3O4 Magnetic Nanoparticles as Peroxidase Mimetics and Their Applications in H2O2 and Glucose Detection. *Anal. Chem.* **80,** 2250–2254 (2008).
31. Cherevko, S. & Chung, C.-H. Gold nanowire array electrode for non-enzymatic voltammetric and amperometric glucose detection. *Sensors Actuators B Chem.* **142,** 216–223 (2009).
32. Ramadoss, K. *et al.* Sign reversal of magnetoresistance in a perovskite nickelate by electron doping. *Phys. Rev. B* **94,** 235124 (2016).
33. Perdew, J. P., Burke, K. & Ernzerhof, M. Generalized gradient approximation made simple. *Phys. Rev. Lett.* **77,** 3865 (1996).
34. Wang, C. *et al.* A facile electrochemical sensor based on reduced graphene oxide and Au nanoplates modified glassy carbon electrode for simultaneous detection of ascorbic acid, dopamine and uric acid. *Sensors Actuators B Chem.* **204,** 302–309 (2014).
35. Huang, Q. *et al.* A sensitive and reliable dopamine biosensor was developed based on the Au@carbon dots–chitosan composite film. *Biosens. Bioelectron.* **52,** 277–280 (2014).
36. Yang, Y. J. & Li, W. CTAB functionalized graphene oxide/multiwalled carbon nanotube composite modified electrode for the simultaneous determination of ascorbic acid, dopamine, uric acid and nitrite. *Biosens. Bioelectron.* **56,** 300–306 (2014).
37. Yuan, D., Chen, S., Yuan, R., Zhang, J. & Liu, X. An ECL sensor for dopamine using reduced graphene oxide/multiwall carbon nanotubes/gold nanoparticles. *Sensors Actuators B Chem.* **191,** 415–420 (2014).
38. Numan, A., Shahid, M. M., Omar, F. S., Ramesh, K. & Ramesh, S. Facile fabrication of cobalt oxide nanograin-decorated reduced graphene oxide composite as ultrasensitive

platform for dopamine detection. *Sensors Actuators B Chem.* **238,** 1043–1051 (2017).
39. Du, J. *et al.* Novel graphene flowers modified carbon fibers for simultaneous determination of ascorbic acid, dopamine and uric acid. *Biosens. Bioelectron.* **53,** 220–224 (2014).
40. Al-Graiti, W. *et al.* Probe Sensor Using Nanostructured Multi-Walled Carbon Nanotube Yarn for Selective and Sensitive Detection of Dopamine. *Sensors* **17,** 884 (2017).
41. Rui, Z. *et al.* Facile synthesis of graphene/polypyrrole 3D composite for a high-sensitivity non-enzymatic dopamine detection. *J. Appl. Polym. Sci.* **134,** 44840 (2017).
42. Tseng, T. T.-C. & Monbouquette, H. G. Implantable microprobe with arrayed microsensors for combined amperometric monitoring of the neurotransmitters, glutamate and dopamine. *J. Electroanal. Chem.* **682,** 141–146 (2012).
43. Khudaish, E. A. *et al.* Sensitive and selective dopamine sensor based on novel conjugated polymer decorated with gold nanoparticles. *J. Electroanal. Chem.* **761,** 80–88 (2016).
44. Li, X., Lu, X. & Kan, X. 3D electrochemical sensor based on poly(hydroquinone)/gold nanoparticles/nickel foam for dopamine sensitive detection. *J. Electroanal. Chem.* **799,** 451–458 (2017).
45. Azadbakht, A., Roushani, M., Abbasi, A. R. & Derikvand, Z. Design and characterization of electrochemical dopamine–aptamer as convenient and integrated sensing platform. *Anal. Biochem.* **507,** 47–57 (2016).
46. Bahrami, S., Abbasi, A. R., Roushani, M., Derikvand, Z. & Azadbakht, A. An electrochemical dopamine aptasensor incorporating silver nanoparticle, functionalized carbon nanotubes and graphene oxide for signal amplification. *Talanta* **159,** 307–316 (2016).
47. Liu, L., Xia, N., Meng, J.-J., Zhou, B.-B. & Li, S.-J. An electrochemical aptasensor for sensitive and selective detection of dopamine based on signal amplification of electrochemical-chemical redox cycling. *J. Electroanal. Chem.* **775,** 58–63 (2016).
48. Wang, W., Wang, W., Davis, J. J. & Luo, X. Ultrasensitive and selective voltammetric aptasensor for dopamine based on a conducting polymer nanocomposite doped with graphene oxide. *Microchim. Acta* **182,** 1123–1129 (2015).
49. Yaqiong Xu, Hun, X., Liu, F., Wen, X. & Luo, X. Aptamer biosensor for dopamine based on a gold electrode modified with carbon nanoparticles and thionine labeled gold nanoparticles as probe. *Microchim. Acta* **182,** 1797–1802 (2015).
50. Rithesh Raj, D., Prasanth, S., Vineeshkumar, T. V & Sudarsanakumar, C. Surface plasmon resonance based fiber optic dopamine sensor using green synthesized silver nanoparticles. *Sensors Actuators B Chem.* **224,** 600–606 (2016).
51. Salamon, J. *et al.* One-pot synthesis of magnetite nanorods/graphene composites and its catalytic activity toward electrochemical detection of dopamine. *Biosens. Bioelectron.* **64,** 269–276 (2015).
52. Jiang, G. *et al.* Application of a mercapto-terminated binuclear Cu(II) complex modified Au electrode to improve the sensitivity and selectivity for dopamine detection. *Sensors Actuators B Chem.* **209,** 122–130 (2015).
53. Gao, G., Zhang, Z., Wang, K., Yuan, Q. & Wang, X. One-pot synthesis of dendritic Pt3Ni nanoalloys as nonenzymatic electrochemical biosensors with high sensitivity and selectivity for dopamine detection. *Nanoscale* **9,** 10998–11003 (2017).
54. Huang, Y., Miao, Y.-E., Ji, S., Tjiu, W. W. & Liu, T. Electrospun Carbon Nanofibers Decorated with Ag–Pt Bimetallic Nanoparticles for Selective Detection of Dopamine. *ACS Appl. Mater. Interfaces* **6,** 12449–12456 (2014).
55. Vijayaraghavan, T., Sivasubramanian, R., Hussain, S. & Ashok, A. A Facile Synthesis of

LaFeO3-Based Perovskites and Their Application towards Sensing of Neurotransmitters. *ChemistrySelect* **2,** 5570–5577 (2017).

56. Li, J., Zhao, J. & Wei, X. A sensitive and selective sensor for dopamine determination based on a molecularly imprinted electropolymer of o-aminophenol. *Sensors Actuators B Chem.* **140,** 663–669 (2009).
57. Li, B. *et al.* Highly selective and sensitive determination of dopamine by the novel molecularly imprinted poly(nicotinamide)/CuO nanoparticles modified electrode. *Biosens. Bioelectron.* **67,** 121–128 (2015).
58. Qian, T. *et al.* Ultrasensitive dopamine sensor based on novel molecularly imprinted polypyrrole coated carbon nanotubes. *Biosens. Bioelectron.* **58,** 237–241 (2014).
59. Teng, Y., Liu, F. & Kan, X. Voltammetric dopamine sensor based on three-dimensional electrosynthesized molecularly imprinted polymers and polypyrrole nanowires. *Microchim. Acta* **184,** 2515–2522 (2017).
60. Baluta, S., Cabaj, J. & Malecha, K. Neurotransmitters detection using a fluorescence-based sensor with graphene quantum dots. *Opt. Appl.* **47,** 225–231 (2017).
61. Gupta, A. & Nandi, C. K. PC12 live cell ultrasensitive neurotransmitter signaling using high quantum yield sulphur doped carbon dots and its extracellular Ca2+ ion dependence. *Sensors Actuators B Chem.* **245,** 137–145 (2017).
62. Kim, H. M. *et al.* Miniaturized and Wireless Optical Neurotransmitter Sensor for Real-Time Monitoring of Dopamine in the Brain. *Sensors* **16,** 1894 (2016).
63. Kruss, S. *et al.* Neurotransmitter Detection Using Corona Phase Molecular Recognition on Fluorescent Single-Walled Carbon Nanotube Sensors. *J. Am. Chem. Soc.* **136,** 713–724 (2014).
64. Zhang, S., Wang, N., Yu, H., Niu, Y. & Sun, C. Covalent attachment of glucose oxidase to an Au electrode modified with gold nanoparticles for use as glucose biosensor. *Bioelectrochemistry* **67,** 15–22 (2005).
65. Wisdom, G. B. Conjugation of Antibodies to Horseradish Peroxidase. *Immunochem. Protoc.* **295,** 127–130 (2005).
66. Gruverman, A. *et al.* Tunneling Electroresistance Effect in Ferroelectric Tunnel Junctions at the Nanoscale. *Nano Lett.* **9,** 3539–3543 (2009).
67. Jaramillo, R., Ha, S. D., Silevitch, D. M. & Ramanathan, S. Origins of bad-metal conductivity and the insulator–metal transition in the rare-earth nickelates. *Nat. Phys.* **10,** 304–307 (2014).
68. Sachtler, W. M. H., Dorgelo, G. J. H. & Holscher, A. A. The work function of gold. *Surf. Sci.* **5,** 221–229 (1966).
69. Yan, F. *et al.* Local charge writing in epitaxial SmNiO3 thin films. *J. Mater. Chem. C* **2,** 3805–3811 (2014).
70. Lee, S.-S., Kim, B. & Lee, S. Structures and Bonding Properties of Gold–Arg-Cys Complexes: DFT Study of Simple Peptide-Coated Metal. *J. Phys. Chem. C* **118,** 20840–20847 (2014).
71. Muir, J. M. R., Costa, D. & Idriss, H. DFT computational study of the RGD peptide interaction with the rutile TiO2 (110) surface. *Surf. Sci.* **624,** 8–14 (2014).
72. Nonella, M. & Seeger, S. Investigating Alanine–Silica Interaction by Means of First-Principles Molecular-Dynamics Simulations. *ChemPhysChem* **9,** 414–421 (2008).
73. Fajín, J. L. C., Gomes, J. R. B. & Cordeiro, M. N. D. S. DFT Study of the Adsorption of d-(l-)Cysteine on Flat and Chiral Stepped Gold Surfaces. *Langmuir* **29,** 8856–8864 (2013).

74. Buimaga-Iarinca1, L. & Calborean, A. Electronic structure of the ll-cysteine dimers adsorbed on Au(111): a density functional theory study. *Phys. Scr.* **86,** 035707 (2012).
75. Rimola, A., Costa, D., Sodupe, M., Lambert, J.-F. & Ugliengo, P. Silica Surface Features and Their Role in the Adsorption of Biomolecules: Computational Modeling and Experiments. *Chem. Rev.* **113,** 4216–4313 (2013).
76. Jimenez-Izal, E., Chiatti, F., Corno, M., Rimola, A. & Ugliengo, P. Glycine Adsorption at Nonstoichiometric (010) Hydroxyapatite Surfaces: A B3LYP Study. *J. Phys. Chem. C* **116,** 14561–14567 (2012).
77. Arrouvel, C., Diawara, B., Costa, D. & Marcus, P. DFT Periodic Study of the Adsorption of Glycine on the Anhydrous and Hydroxylated (0001) Surfaces of α-Alumina. *J. Phys. Chem. C* **111,** 18164–18173 (2007).
78. Carravetta, V., Monti, S. & Zhang, W. Interaction of biomolecular systems with titanium-based materials: computational investigations. *Theor. Chem. Acc.* **123,** 299–309 (2009).
79. Vanommeslaeghe, K. *et al.* CHARMM general force field: A force field for drug-like molecules compatible with the CHARMM all-atom additive biological force fields. *J. Comput. Chem.* **31,** 671–690 (2009).
80. MacKerell, A. D. *et al.* All-Atom Empirical Potential for Molecular Modeling and Dynamics Studies of Proteins. *J. Phys. Chem. B* **102,** 3586–3616 (1998).
81. Rappe, A. K., Casewit, C. J., Colwell, K. S., Goddard, W. A. & Skiff, W. M. UFF, a full periodic table force field for molecular mechanics and molecular dynamics simulations. *J. Am. Chem. Soc.* **114,** 10024–10035 (1992).
82. Kresse, G. & Furthmüller, J. Efficient iterative schemes for ab initio total-energy calculations using a plane-wave basis set. *Phys. Rev. B* **54,** 11169 (1996).
83. Kresse, G. & Joubert, D. From ultrasoft pseudopotentials to the projector augmented-wave method. *Phys. Rev. B* **59,** 1758 (1999).
84. Liechtenstein, A. I., Anisimov, V. I. & Zaanen, J. Density-functional theory and strong interactions: Orbital ordering in Mott-Hubbard insulators. *Phys. Rev. B* **52,** R5467 (1995).
85. Allen, M. P. & Tildesley, D. J. *Computer simulation of liquids*. (Oxford university press, 1989).
86. Wu (吴秋雨), Q. *et al.* Integration of autopatching with automated pipette and cell detection in vitro. *J. Neurophysiol.* **116,** 1564–1578 (2016).
