# Supplementary material for: Perovskite nickelates as bio-electronic interfaces
Source: Nat Commun. 2019 Apr 10;10:1651. doi: 10.1038/s41467-019-09660-6 (PMC6458181; doi:10.1038/s41467-019-09660-6)
Supplement: Supplementary file 2 — Description of Additional Supplementary Files [file 41467_2019_9660_MOESM2_ESM.pdf]

Description of Additional Supplementary Files

File Name: Supplementary Movie 1
Description: **MD simulation trajectory showing the interaction of SNO with gluconolactone and FADH2.** MD trajectory highlights the conformal dynamics of FADH2 before it approaches the SNO surface. The spheres here represent nickel (green), samarium (purple), oxygen (red), nitrogen (blue), carbon (grey) and hydrogen (white).

File Name: Supplementary Movie 2
Description: **AIMD movie showing the spontaneous hydrogen transfer mechanism.** The hydrogen transfer is facile and occurs within a fraction of a picosecond. Note that the SNO slab is held fixed to reduce the computational cost. The spheres here represent nickel (green), samarium (purple), oxygen (red), nitrogen (blue), carbon (grey) and hydrogen (white)

File Name: Supplementary Movie 3
Description: **AIMD movie showing the absence of hydrogen transfer due to steric effects.** The conformation of the FADH2 molecule plays a key role in dictating the hydrogen transfer. As one example, in this movie, we observe that the molecule undergoes conformational change and does not dehydrogenate with the AIMD timescales. Note that the SNO slab is held fixed to reduce the computational cost. The spheres here represent nickel (green), samarium (purple), oxygen (red), nitrogen (blue), carbon (grey) and hydrogen (white).
